# Toxicological effects of micro/nano-plastics on mouse/rat models: a systematic review and meta-analysis

**DOI:** 10.3389/fpubh.2023.1103289

**Published:** 2023-05-18

**Authors:** Weijia Liu, Bowen Zhang, Qianqian Yao, Xihua Feng, Tianling Shen, Peisen Guo, Panpan Wang, Yitong Bai, Bo Li, Peixi Wang, Ruiling Li, Zhi Qu, Nan Liu

**Affiliations:** ^1^Institute of Environment and Health, South China Hospital of Shenzhen University, Shenzhen, China; ^2^Institute of Chronic Disease Risks Assessment, School of Nursing and Health, Henan University, Kaifeng, China; ^3^College of Public Health, Zhengzhou University, Zhengzhou, China

**Keywords:** micro/nano-plastics (MNPs), toxic effects, environmental toxicology, biological endpoints, health risk

## Abstract

Micro/nano-plastics (MNPs) are considered a heterogeneous class of environmental contaminants that cause multiple toxic effects on biological species. As the commonly used mammalian models to study the effects of MNPs with regard to their toxic effects, the mouse and rat models are making a great contribution to the disciplines of environmental toxicology and medical health. However, the toxic effects of MNPs have not been systematically summarized. Therefore, a systematic review and a meta-analysis of the toxic effects of MNPs on mouse/rat models were conducted. A total of seven main categories were established in this systematic review, and 24 subcategories were further divided according to the specific physiological significance of the endpoint or the classification of the physiological system, which covered all the selected pieces of literature. A total of 1,762 biological endpoints were found, and 52.78% of them were significantly affected. This fact indicates that there are relative factors, including the size, polymer type, concentration, and exposure time of MNPs and different sexes of mouse/rat models that could significantly affect the biological endpoints. These biological endpoints can be classified into various factors, such as the dose–response relationships between MNP concentration and physiological categories of the nervous system, growth, reproduction, digestive tract histopathology, and inflammatory cytokine level, among others. MNPs negatively affected the blood glucose metabolism, lipid metabolism, and reproductive function in mice. The reproductive function in male mice is more sensitive to the toxic effects of MNPs. These findings also provide insights into and directions for exploring the evidence and mechanisms of the toxic effects of MNPs on human health. It is clear that more research is required on the pathological mechanisms at the molecular level and the long-term effects of tissue accumulation.

## 1. Introduction

As the demand for plastic products has increased in recent years, global production of plastics has reached nearly 368 million metric tons, and improper disposal of plastic waste could lead to long-term exposure to environmental media (1). As one of the emerging pollutants, micro/nano-plastics (MNPs), with particle sizes of <5 mm and 1 μm, respectively (2, 3), whether derived directly from industrial, medical, and household products or from the larger plastics degradation, are currently receiving more worldwide attention. Another additional concern is that the ubiquity of microplastic contamination creates numerous ecological and environmental problems, as well as health issues for humans and other species. The three main ways of human intake of MNPs include the digestive tract, the respiratory tract, and skin contact (4). Many types of evidence have confirmed that humans continue to suffer from MNP pollution ever since the discovery by Thomson (5–11). Humans are getting exposed to MNPs through the inhalation of particles in the air and ingestion in diet, water, and dust (9, 12, 13). Moreover, it has been demonstrated that an infant is at a greater risk of MP exposure than an adult (14). At present, the accumulation of MNPs has been found in a variety of edible organisms, including in some foods taken daily and drinking water, as well as honey and tap water. In addition, mammals can take up more MNPs through biomagnification (15). At the same time, atmospheric particulate matter also contains a large amount of MNPs (11). The number of MPs inhaled through the respiratory tract can reach 553 per day, and this intake can irreversibly accumulate to 8.32 × 10^3^ (16). With the current global COVID-19 pandemic, wearing a mask has become an indispensable part of human daily life, while it is also found to be one of the sources of human intakes of MNPs (17–19). A recent survey found that a single mask releases more than 1 billion MNPs into the environment. MNPs have been found in human placenta, stools, lung tissue, human breastmilk, and blood, among others (8, 13, 20–24). This indicates that emerging environmental pollutants are having a long-term impact on human health and are becoming a global problem and concern.

Although the above evidence demonstrated the continuous exposure of MNPs to living species and human beings, the toxicological effects of MNPs still remain unknown (1, 4, 6, 25, 26). Based on medical ethics, we cannot impose experimental and environmental contaminants on humans. Consideration should be given to the extensive scientific exploration and observations that were obtained from *in vitro* studies (27) and animals, including mammals (28, 29).

Owing to their characteristics, terrestrial mammals were the common animal models for the exploration of the toxicological effects of MNPs. Most of the studies in this field have focused on mice and rats, and they have both made remarkable contributions to the progress of biomedicine (30, 31). Several studies have reported that exposure to MNPs could cause oxidative stress and inflammatory reactions (32–39). Nevertheless, other studies have found no effects of long-term low-dose exposure to MNPs on the mouse/rat models (40). These findings indicate that differences in plastic-type, exposure time, and concentration can result in differences in toxicological effects. The toxicological mechanism of MNPs still needs to be explored further (41).

We collected data to conduct a systematic analysis of the toxicological effects of MNPs on mouse/rat models and infer the toxicological effects on humans. Based on this study, we investigated the effects of different MNP types and exposure time on metabolism and reproduction in mice. We also explored the differences in reproductive toxicity of MPs in mice with different sexes. In addition, we performed a meta-analysis to investigate the effects of exposure to MNPs on glucose metabolism, reproduction, oxidative stress, and lipid metabolism in mice with sufficient data for predicting the impact of MNPs on human metabolism and reproduction. Our research supports the control of plastic pollution, including the reduction in plastic production, a gradual introduction of more environment-friendly and harmless alternatives, strict control of diet, and MNPs levels in the air. The present study aimed to provide a reference for speculating on the health effects of MNPs on humans; meanwhile, it also provides some evidence for conducting animal experiments.

## 2. Materials and methods

### 2.1. Study design

The study was conducted following the Preferred Reporting Items for Systematic Reviews and Meta-Analyses (PRISMA) guidelines.

### 2.2. Literature retrieval strategy

We conducted the literature search through four universally used English electronic bibliographic databases, PubMed, Web of Science, ScienceDirect, and Springer databases, and two Chinese academic databases, China National Knowledge Infrastructure (CNKI) and Wanfang, using the term “Microplastic^*^ or Nanoplastic^*^ or Plastic particles or Polyethylene microplastic^*^ or Polyvinylchloride microplastic^*^ or Polypropylene microplastic^*^ or Polystyrene microplastic^*^ or Polyamide microplastic^*^” and “Mice or Rat^*^” from inception to 28 February 2022.

### 2.3. Eligibility criteria

**Figure 1:**
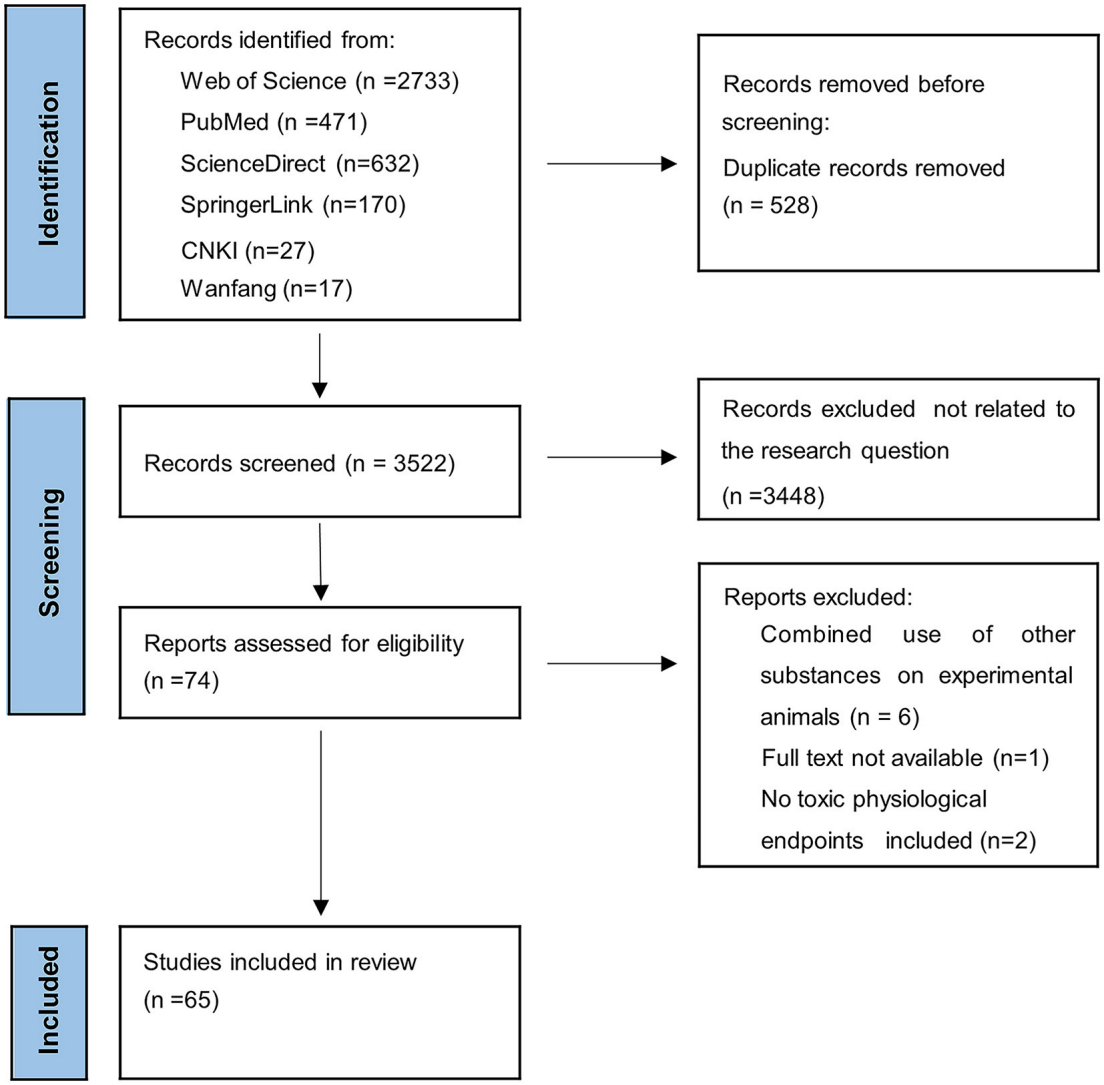
Modified Preferred Reporting Items for Systematic Reviews and Meta-Analysis (PRISMA) flow diagram of the literature search and selection strategy.

The search was not restricted to any language. The inclusion criteria of the present study were as follows: (1) Exposure to pristine MNPs based on the laboratory conditions of a mouse/a rat, (2) at least one toxicological physiological endpoint included in the findings, and (3) a blank control group was set up. The exclusion criteria were: (1) the effects of other substances on laboratory animals; (2) a lack of appropriate data in the study; (3) low-quality studies assessed using the Rob tool for animal intervention studies (SYRCLE's Rob tool) (42); and (4) unavailability of full-text articles. Two authors (WL and BZ) independently searched the database and decided to include or exclude publications according to the criteria established above. Inconsistencies were resolved through discussion and consultation with the third author (QY). The detailed Modified Preferred Reporting Items for Systematic Reviews and Meta-Analysis (PRISMA flow diagram for the literature search and selection strategy is exhibited in Figure 1.

### 2.4. Systematic review

#### 2.4.1. Extraction of information

The following information was extracted from the final included studies: published year of the study, species (mice and rat) and sex (male and female) of the experimental animals, the size and concentration of MNPs, polymer type [polystyrene (PS), polyethylene (PE), and polypropylene (PP)], and exposure time.

##### 2.4.1.1. Biological endpoints and classifications

All the biological endpoints were classified according to the research significance of each study. We recorded all data if a study included multiple MNP treatments with different concentrations, sizes, or forms for one endpoint. If a biological endpoint was measured by multiple measurements throughout a continuous exposure of the same experimental animal in the study, the event would be determined based on the final result. Simultaneously, if it indicated more than one biological significance, we would categorize the endpoints according to the purpose of the study. Moreover, if an endpoint was significantly different in the study relative to the control group, indicating that this endpoint was affected, we marked it as “YES”.

A total of seven main categories were established in this systematic review, and 24 subcategories were further divided according to the specific physiological significance of the endpoint or the classification of the physiological system, which covered all the selected pieces of literature. The proportion affected for each category or subcategory was defined as the number of endpoints affected divided by the number of all endpoints in that category or subcategory. The specific classifications of biological endpoints are listed in Table 1.

**Table 1:** Specific classification of biological endpoints.

**Categories**	**Subcategories**	**Index**
Behavioral, sensory, and neuromuscular functions	Activity and locomotion	Social interactions
	Consumption	Food consumption
	Muscular functions	Change in muscle weight
	Nervous system	PD-like neurodegeneration
	Sensory perception	Anxiety
Fitness	Development of pups	Development of pups
	Growth	Change in body weight
	Reproduction investment and success	Sperm count
	Survival	Survival rate
Gut system	Digestive tract histopathology	Inflammation score
	Intestinal permeability and barrier function	Intestinal mucosal layer
	Gut microbiota	Actinomycetes
	Stool parameters	Stool number
Hematopoietic system	Hematopoiesis	Colony-forming unit-granulocyte and macrophage
	Hematological changes	Complete blood cell counts
Immune system	Change in immune cells	Eosinophils
	Inflammatory cytokine level	Ig E
Metabolism	Amino acids metabolism	Arginine content
	Carbohydrate metabolism	Plasma glucose
	Detoxification	Glutathione content
	Hormone and trace elements levels	Follicle-stimulating hormone
	Lipid metabolism	Triglycerides content
	Oxidative stress	Catalase activity
Visceral organ injury	Organ histopathology	Liver injury

#### 2.4.2. Concentration classification of MNPs exposure

To explore the potential effects of different concentrations and doses of MNPs on a mouse and a rat, we divided the endpoints into different levels of concentration and dose. Different concentration units used in different studies were dependent on the ways of administration. We arbitrarily established different levels of concentration and dose to keep the endpoint distribution in different classes uniform. Then, we established four levels of concentration in studies administered *via* drinking water, including concentrations <10 μg L^−1^, 10-100, 100-1000, and ≥ 1000 μg L^−1^ for Class 1-4, respectively. Furthermore, four concentration levels were established in studies by gavage as concentrations <10 mg, 10-100, 100-1000, and ≥1000mg kg^−1^ for Class 1-4, respectively. The grading of MNP doses was chosen as <0.1, 0.1-1, and ≥ 1 mg for Class 1-3, respectively.

#### 2.4.3. Exposure time and size classification for the systematic review

We classified MNPs into two categories based on the definitions of MPs and NPs: sizes <1 μm for NPs and 1 μm ≤ sizes <5 mm for MPs. We established three levels of exposure time in studies to explore its effect on endpoints as follows: ≤ 28 days, 28–84 days, and ≥ 84 days for classes 1-3, respectively.

### 2.5. Meta-analysis

#### 2.5.1. Data extraction

We evaluated all the included literature in systematic reviews with sufficient data, which were employed for meta-analysis. The selected studies were categorized according to classification and biological endpoints. The effects of MNPs on metabolism and reproduction in mice and rats were explored.

To reduce the bias, we excluded an endpoint if it was included by <3 studies. We selected four endpoints with sufficient data for meta-analysis: glucose metabolism (e.g., glucose content in serum), reproduction (e.g., sperm count), oxidative stress (e.g., catalase activity), and lipid metabolism (e.g., triglyceride metabolism) in mice. We extracted relevant information, including sample size and the mean and variance for the treatment and control groups, for each endpoint from the abovementioned selected studies. Data were extracted from the original text, tables, or figures in each study with the software GetData Graph Digitizer 2.26.

#### 2.5.2. Calculation of weighted mean values

Hedges' g was used to calculate the effect size for each endpoint including glucose metabolism/reproduction/oxidative stress/lipid metabolism, which is the bias-corrected standardized mean difference between the treatment and control groups. Hedges' g can be obtained by multiplying Cohen's d and the correction factor J as Equation (1) (43).


(1) $$\begin{array}{l} Hedges^{\prime }\;g\;=\frac{\left(Mean_{T}-Mean_{C}\right)}{\sqrt{\frac{\left(n_{T}-1\right)SD_{T}^{2}+\left(n_{C}-1\right)SD_{C}^{2}}{n_{T}+n_{C}-2}}}\times \left(1-\frac{3}{4\left(n_{T}+n_{C}-9\right)}\right) \end{array}$$

where T and C represent the treatment and control groups, respectively, SD refers to the standard deviation, and n is the sample size. If the standard error (SE) is provided in the study, we can convert through the formula $SD=SE\times \sqrt{n}$ to obtain the required dates.

The variance (V) for each effect size is calculated as Equation (2):


(2) $$\begin{array}{l} V=\frac{n_{T}+n_{C}}{n_{T}n_{C}}+\frac{g^{2}}{2\left(n_{T}+n_{C}\right)} \end{array}$$

Because the environmental conditions and methods of each study were different, we used a random-effects model in the meta-analysis to calculate the average effect size for each indicator, which has been weighted using the inverse of variance (43). We calculated the effect sizes and 95% confidence intervals (CIs) of MNPs for these four categories, and further analysis was carried out using the exposure time, sex selection, and polymer type as moderators, respectively.

#### 2.5.3. Publication bias and sensitivity analysis

Studies included in the meta-analysis were tested for a quantitative assessment of publication bias using funnel plots and Egger's test. The sensitivity of the meta-analysis was assessed according to the included studies by cutting them out one by one.

### 2.6. Statistical analysis

The data were summarized and sorted using Origin 2021 and RStudio with the ggplot2 package (https://www.rstudio.com/) which was used for drawing; in addition, meta and metaphor packages (44) in RStudio were employed for meta-analysis.

## 3. Results

### 3.1. Study characteristics

We obtained 4,050 articles through our search, of which 528 duplicate articles were removed. The remaining 3,522 articles were then screened, of which 3,448 articles unrelated to the target question were excluded and 9 articles that did not meet the pre-established criteria were excluded through a full-text review. Finally, 65 studies were included in this systematic review (Figure 1), which were published between 2017 and 2022 and were mainly conducted in China (*n* = 42) and South Korea (*n* = 11). The basic characteristics of the incorporated literature for meta-analysis are demonstrated in Table 2.

**Table 2:** Basic characteristics of the incorporated literature for meta-analysis.

**First author**	**Year of publication**	**Composition**	**Particle size**	**Physiological endpoint**	**Reference**
Deng Y.	2017	PS	5 μm	Lipid metabolism Oxidative stress	(45)
Fan X.	2022	PS	80 ± 6 nm	Glucose metabolism Oxidative stress	(46)
Han Y.	2021	PE	10-45 μm	Lipid metabolism	(40)
Hou B.	2021	PS	5 μm	Reproduction	(47)
Jiang P.	2021	PS	5 μm	Lipid metabolism	(48)
Jin Y.	2019	PS	5 μm	Lipid metabolism	(49)
Lee S.	2022	PE	10-50 μm	Glucose metabolism	(50)
Li S.	2021	PS	< 5 mm	Glucose metabolism	(51)
Lu L.	2018	PS	0.5 μm 50 μm	Lipid metabolism	(52)
Luo T.	2019	PS	5 μm	Lipid metabolism	(53)
Meng X.	2022	PS	300 nm	Oxidative stress	(36)
Park E.J.	2020	PE	40-48 μm	Reproduction	(54)
Sun H.	2021	PE	1-10 μm	Glucose metabolism Lipid metabolism	(37)
Wang S.	2021	PS	1-10 μm 50-100 μm	Oxidative stress	(55)
Wei Y.	2021	PS	4 and10 μm	Reproduction	(56)
Wei Z.	2022	PS	5-5.9 μm	Reproduction Oxidative stress	(57)
Xie X.	2020	PS	5-5.9 μm	Reproduction Oxidative stress	(39)
Zhao J.	2022	PS	4 and 5 μm	Glucose metabolism	(58)

### 3.2. General effects of physiological functions on the mouse/rat models by MNPs

**Figure 2:**
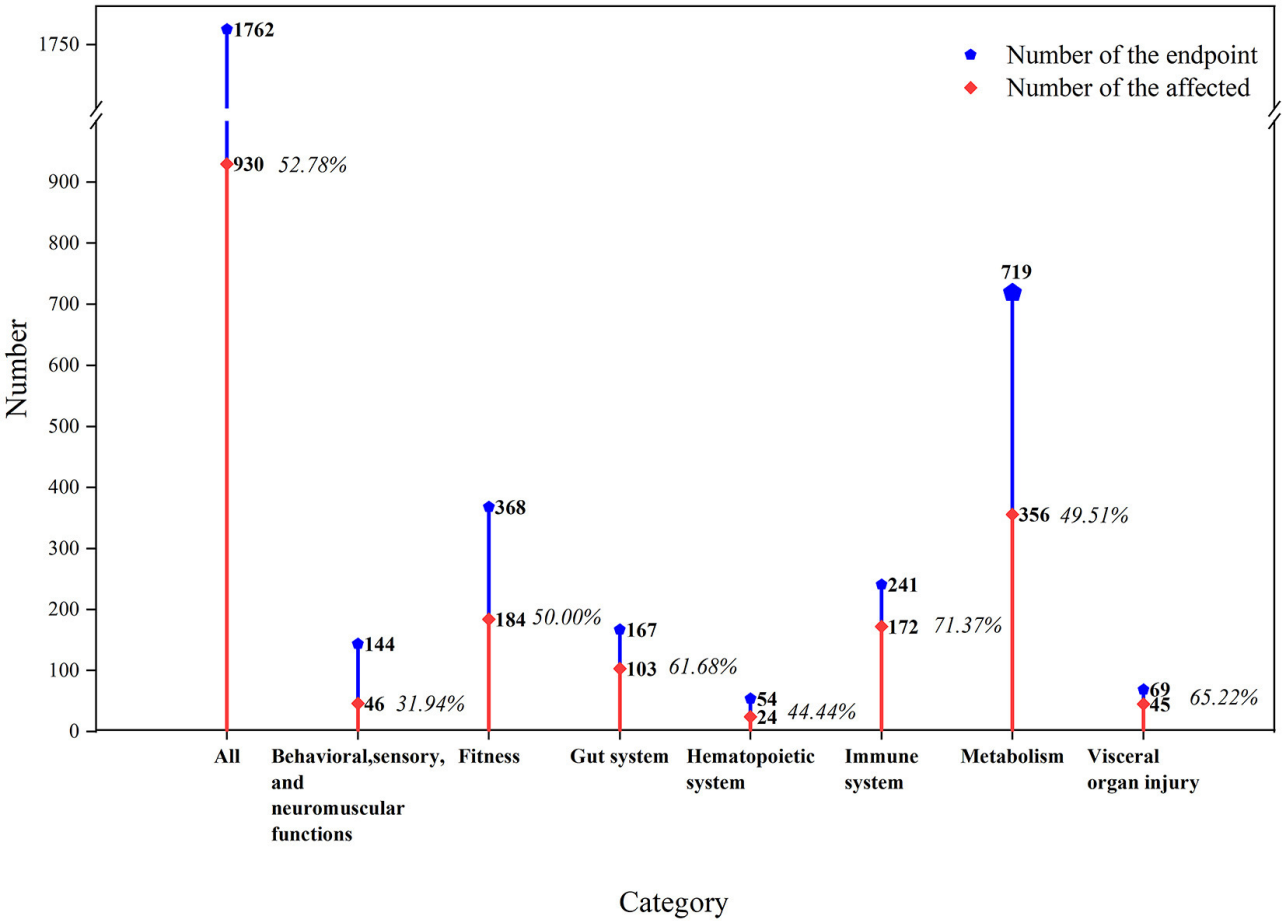
Toxicological effects of MNPs on the biological functions of mouse/rat models. The orange line indicates the proportion of affected biological endpoints (i.e., significant differences between the exposed and non-exposed groups).

We extracted a total of 1,762 biological endpoints from the selected 65 studies given in Supplementary Table S1, including seven main categories as the physiological functions in the mouse/rat models (Figure 2). We observed that 52.78% of biological endpoints were significantly affected by exposure to MNPs. Furthermore, metabolic biological endpoints were discovered to be the most affected endpoints (*n* = 719), with the percentage of endpoints reaching 49.51%. Fitness followed closely with metabolism (*n* = 368), with the percentage of endpoints reaching 50.00%.

**Figure 3:**
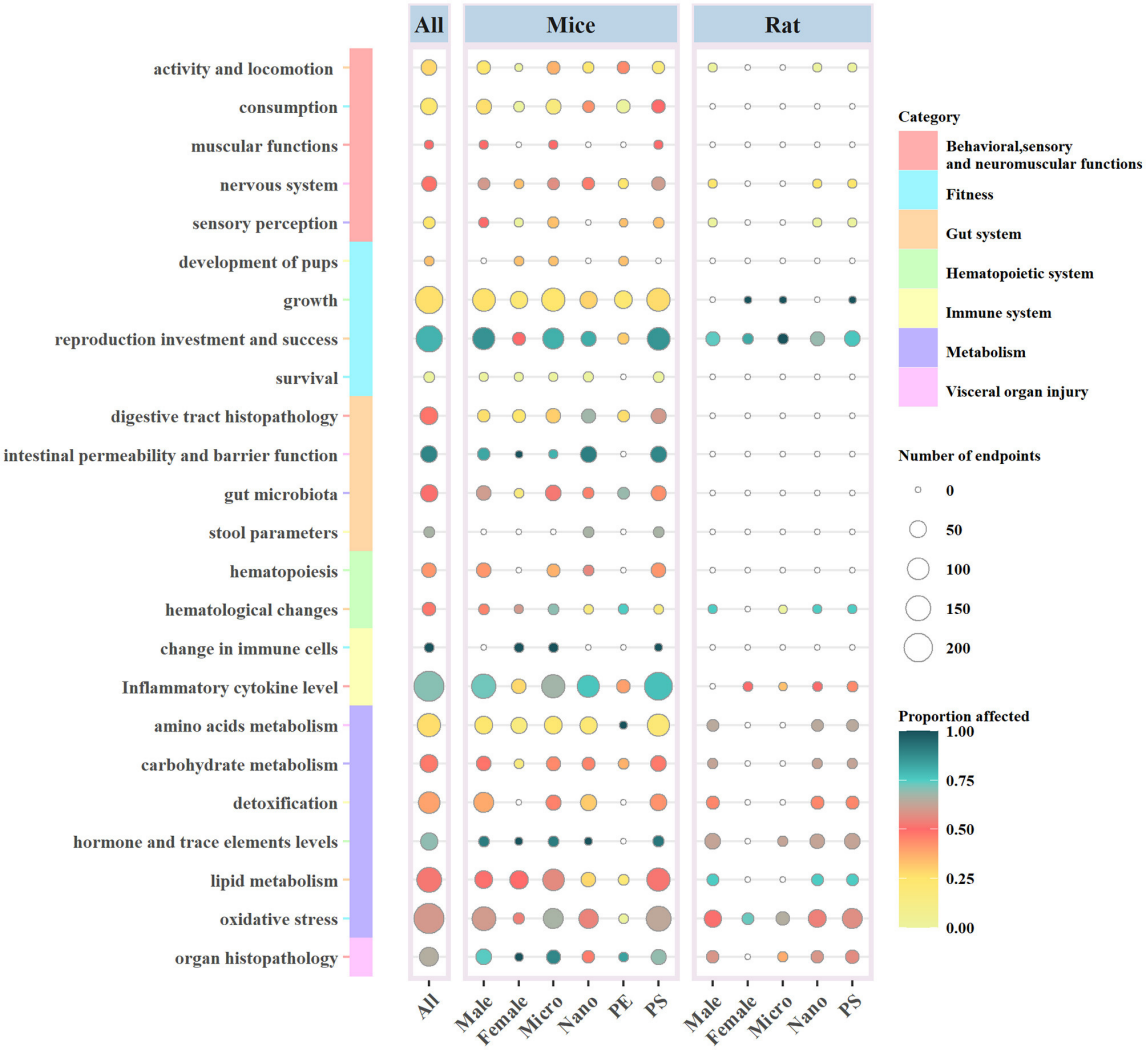
Ecotoxicological effects of MNPs on the biological functions of mouse/rat models according to the taxonomic group, MNP size (MPs or NPs), sex of the experimental animal, and polymer type. Circle size and color indicate the number and proportion of the affected biological endpoints (i.e., significant differences between the exposed and non-exposed groups). PS, polystyrene; PE, polyethylene.

The specific details of the ecotoxicological effects of MNPs on the biological functions of the mouse/rat model are shown in Figure 3.

#### 3.2.1. Behavioral, sensory, and neuromuscular functions

The consumption of food and water had the largest number of endpoints in this category (*n* = 50), with only 24% affected, followed by activity and locomotion (*n* = 39) with a poor proportion of affected endpoints reaching 28.21%. MNPs can become deposited in the brain of animal models and cause neurobehavioral abnormalities. Biological endpoints involving the nervous system in the studies include Parkinson's disease (PD)-like neurodegeneration (59) and learning and memory behavior (60). The number of biological endpoints in the nervous system was only 35, with 48.57% of endpoints affected.

#### 3.2.2. Fitness

In this category, most of the endpoints focused on both growth (*n* = 183) and reproduction (*n* = 168), which were considered to be the most affected with 79.76% of endpoints. Reproductive endpoints were measured differently depending on the sex of the experimental animal, such as the count of sperm and follicle in male (57) and female mice (61). At the same time, although the number of endpoints in growth was the largest, only 26.23% of them affected were placedin this category.

#### 3.2.3. Gut system

The affected biological endpoints of the gut system reached 61.68%, and most of them were focused on intestinal permeability and barrier function. It was reported that MNPs could damage the intestinal mucosa of mice and affect the barrier function of the intestinal system (62, 63). Close attention was paid to changes in gut microbiota by MNPs through the measurement of the diversity (*n* = 53) (48, 64, 65), and 50.94% of the endpoints were affected in this category.

#### 3.2.4. Hematopoietic system

The toxicological effects on the hematopoietic system were displayed in mice, with 44% of the endpoints affected in this category (58, 66, 67).

#### 3.2.5. Immune system

The degree of impact on the immune system (*n* = 241) was the maximum among the seven main categories, with 71.37% of the endpoints being affected, including the damage to immune cells (68, 69) and the abnormal level of secretion of inflammatory cytokines (36, 39).

#### 3.2.6. Metabolism

The metabolic effects of MNPs on the mouse/rat models were mainly concentrated in factors such as oxidative stress (*n* = 236), lipid (*n* = 151), and amino acid metabolism (*n* = 123). Among these factors, most of the endpoints of oxidative stress were affected (59.75%).

#### 3.2.7. Visceral organ injury

MNPs had a significant effect on visceral organ injury in the mouse/rat models (*n* = 69), and 65.22% of the endpoints were significantly affected. Toxicological effects of MNPs could be observed and determined by the pathological sections of the internal organs (46, 70, 71), and there were varying degrees of damage to the target organs, such as the liver, kidney, and lung.

### 3.3. Effects of MNP size on biological endpoints

There were 494 endpoints (52.05%) related to MPs and mouse/rat models significantly affected. The number of biological endpoints for the effect of NPs on models was 813, and 53.63% of them were affected (Figure 3).

Growth (*n* = 128) and inflammatory cytokine level (*n* = 123) were considered as the most noteworthy endpoints by MPs on rats and mice, while the number of endpoints with regard to oxidative stress (*n* = 132) was shown to be the same for both MPs and NPs. MPs and NPs had the most significant effect on intestinal permeability and reproduction, with proportions of 80.00% and 90.48%, respectively. The latter might be the smallest number of endpoints in this area (*n* = 47) (Figure 2).

### 3.4. Effects of the polymer type of MNPs on biological endpoints

The polymer types of MNPs used in the included articles were PE, PP, and PS. It could be observed that PS and PE were the most commonly used MNPs, and there was only one report on the employment of PP. We found 1,464 biological endpoints that were observed for the toxicity of PS. A few studies reported that functional group modifications of carboxyl or amino to PS could change its toxicity, and the results demonstrated that the endpoints were more affected by PS-NH_2_ (65, 72). The number of biological endpoints affected by PS on the inflammatory cytokine level (*n* = 203) and reproduction (*n* = 153) occupied the top two positions, with proportions of 75.86 and 83.66%, respectively. Moreover, PS showed more obvious toxicological effects on reproduction than PE, probably due to the low number of biological endpoints and the less attention given to the use of PE (Figure 3).

### 3.5. Effects on different sexes of mouse/rat models by MNPs

It has been demonstrated that the majority of the biological endpoints (*n* = 1193) in the selected experimental mouse/rat models were males, and only 340 were females. The overwhelming biological endpoints for females were focused on lipid and amino acid metabolism and growth. Moreover, male fertility was more susceptible to MNPs than female fertility, accounting for 83.94% of the mouse/rat models affected with reproductive endpoints (Figure 3).

### 3.6. Effects of different concentrations of MNPs on biological endpoints

**Figure 4:**
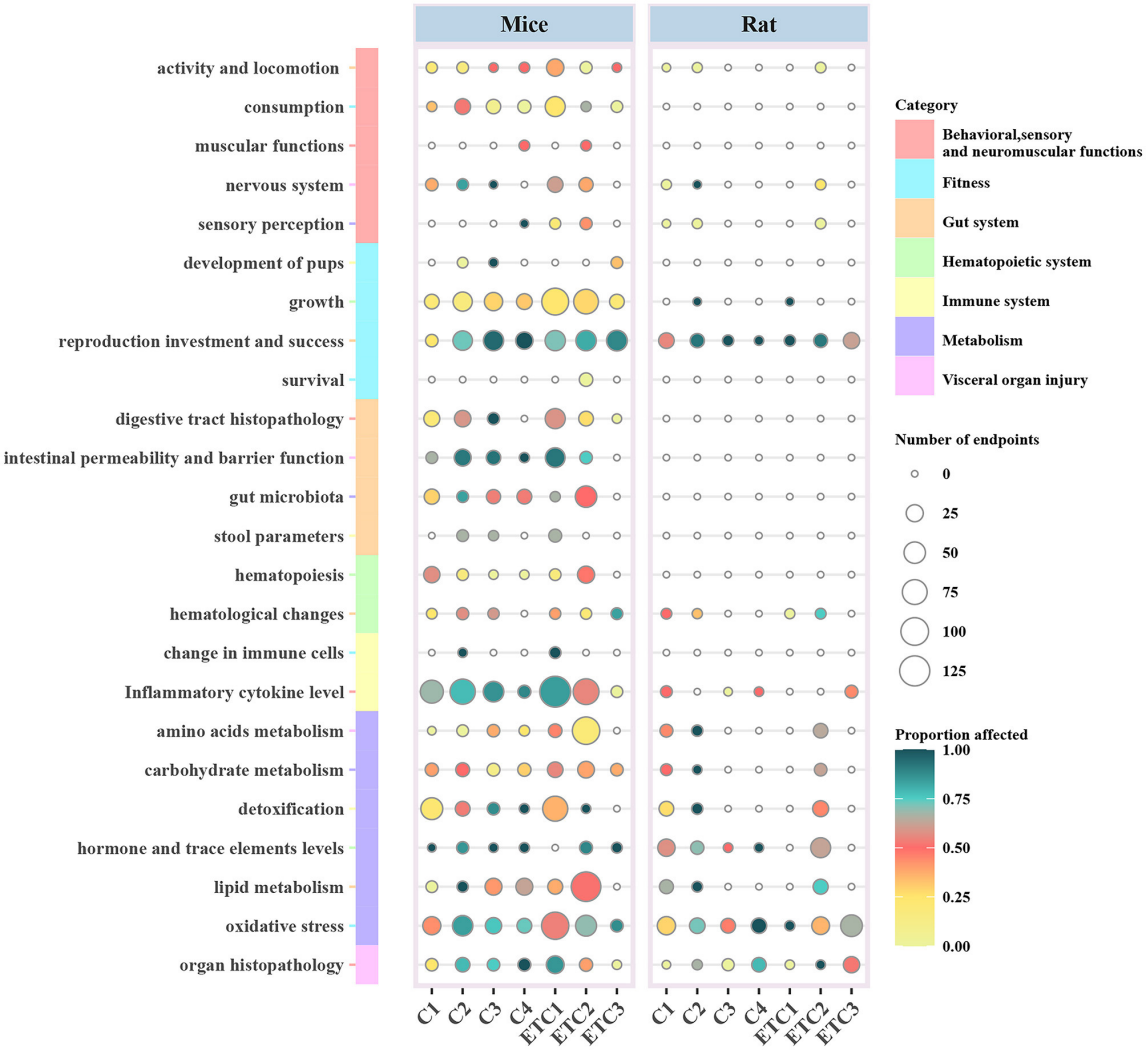
Ecotoxicological effects of MNPs on the biological functions of mouse/rat models according to the classification of concentrations. (Concentrations <10 μg L^−1^, 10-100, 100-1000, and ≥ 1000 μg L^−1^ for Class 1-4, respectively). Furthermore, four concentration levels were established by gavage as Class 1-4 with the same concentrations, respectively. The grading of MNP doses was chosen as <0.1, 0.1-1, and ≥ 1 mg for Class 1-3, respectively; and the exposure time classes were divided into ≤ 28, 28-84, and ≥ 84 days for Class 1-3, respectively. Circle size and color indicate the number and proportion of the affected biological endpoints (i.e., significant differences between the exposed and non-exposed groups). C1-C4, Concentration class 1-4; ETC, exposure time classes.

We observed a positive relationship between the number of affected biological endpoints and the concentrations of MNPs (Figure 4). Compared with Classes 1 and 2, the proportions of biological endpoints were relatively higher in Classes 3 and 4 with proportions of 62.76 and 67.84%. There was a clear positive dose–response relationship between the concentration of MNPs and toxicological effects on the exposed organisms. Particular evidence has been confirmed in the physiological categories of the nervous system, reproduction, digestive tract histopathology, and inflammatory cytokine level (Figure 4).

### 3.7. Effects of different exposure times on biological endpoints

A large proportion (59.91%) of biological endpoints were affected by a relatively prolonged exposure time of Class 3 compared with those of Classes 1 and 2, especially with regard to hematological changes, detoxification, and lipid metabolism. Whereas, other biological endpoints were less affected, possibly due to the relatively lower concentrations of MNPs.

### 3.8. Meta-analysis

**Figure 5:**
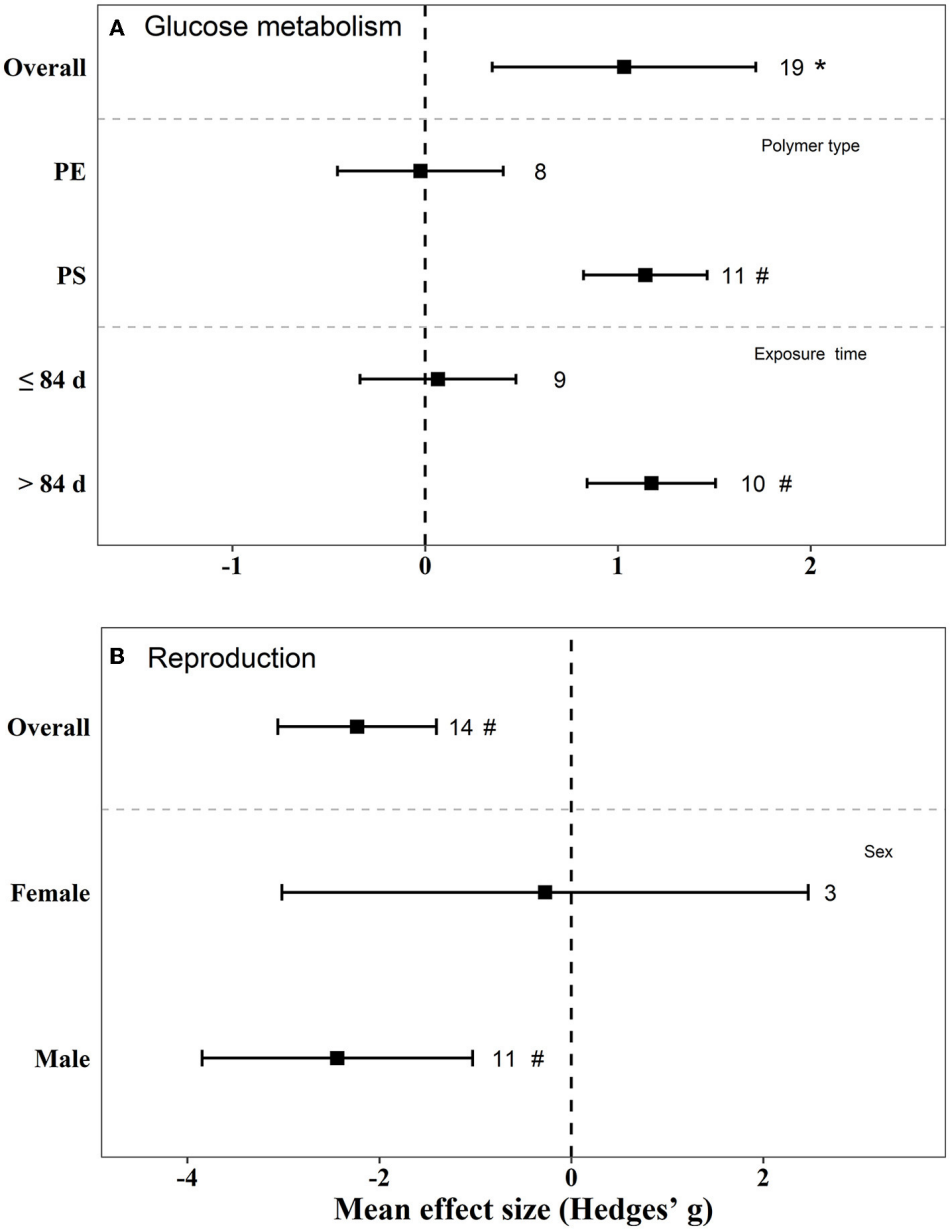
Mean effect sizes ±95% CI for glucose metabolism and reproduction in mice. Effect sizes calculated as Hedges' g. Sample sizes are noted beside bars. Differences that are statistically significant (*p* < 0.05 and 0.01) are marked with * and ^#^.

**Figure 6:**
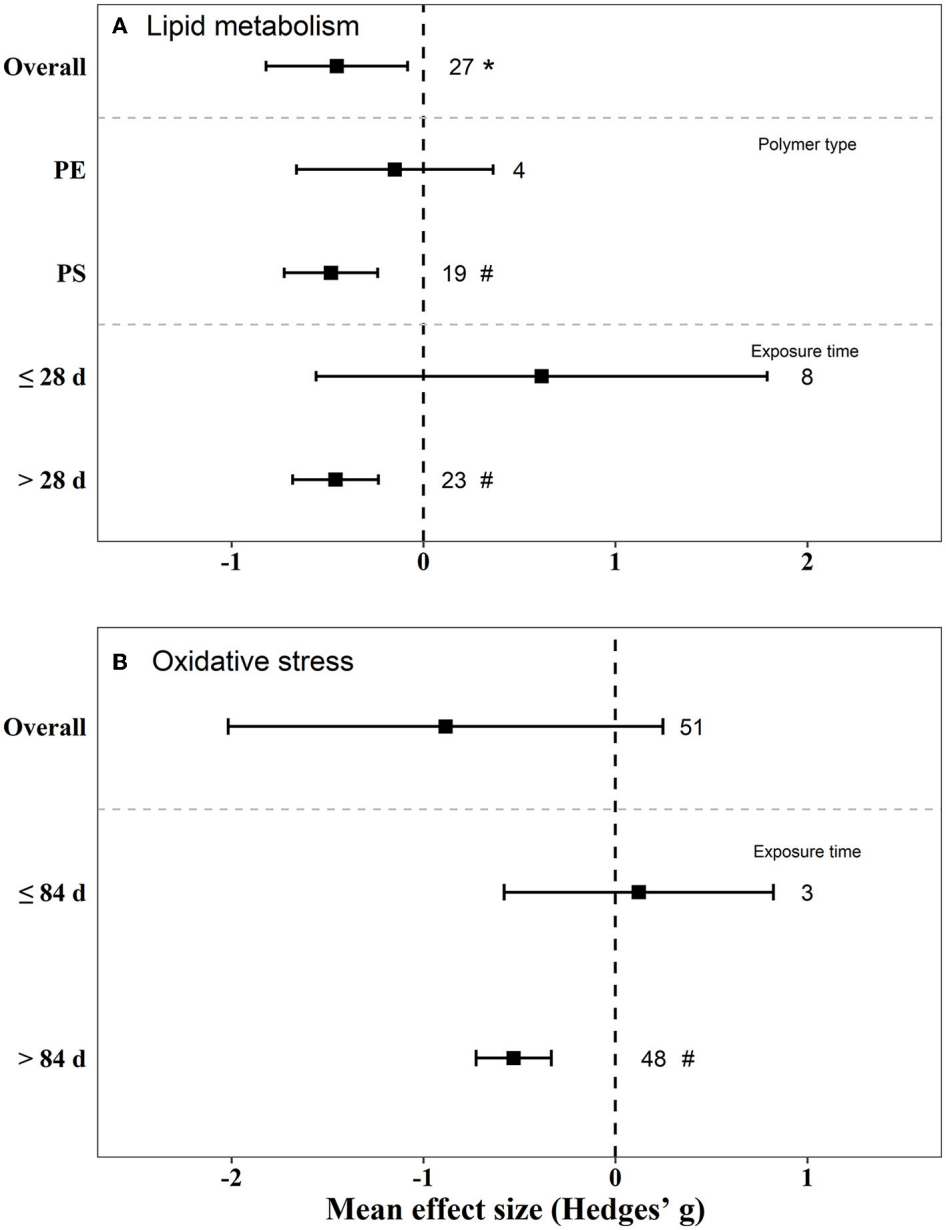
Mean effect sizes ±95% CI for lipid metabolism and oxidative stress in mice. Effect sizes are calculated as Hedges' g. Sample sizes are noted beside bars. Differences that are statistically significant (*p* < 0.05 and 0.01) are marked with * and ^#^.

There were negative effects on reproduction (Hedges' g = −2.23, 95% CI: −3.06–1.41), glucose metabolism (Hedges' g = 1.03, 95% CI: 0.35–1.72), and lipid metabolism (Hedge” g = −0.45, 95% CI: −0.82 to −0.08) in mice by exposure to MPs (Figures 5, 6). We also found that, when the exposure time was >3 m, the biological endpoints of mice, such as blood glucose metabolism and oxidative stress, changed. Furthermore, male mice were more sensitive to the reproductive toxicity of MNPs than female mice.

The effect of PS on blood glucose and lipid metabolism in mice was greater than that of PE (Figures 5, 6). We also tested the independent publication bias for these four responses using funnel plots, and Egger's tests were used to assess asymmetry (Supplementary Figures S1–S4). The results suggested asymmetry for glucose metabolism (*z* = 2.64, *p* = 0.017), reproductive effects (*z* = −5.30, *p* < 0.001), oxidative stress (*z* = 3.32, *p* = 0.002), and lipid metabolism (*z* = −2.12, *p* = 0.040) in mice. Different groups of concentration and exposure time were the main reasons for the asymmetric distribution of the funnel plot. Sensitivity analysis of effect size revealed no significant difference for each data set (Supplementary Figures S5–S8).

## 4. Discussion

It was reported that MPs were toxic to soil invertebrates and marine mammals (73, 74); however, a systematic summary of the toxicological effects of MNPs on terrestrial mammals, including mouse and rat, is lacking. Mouse and rat are the models and representatives of terrestrial mammals in the world. As the common and traditional model organisms in environmental toxicology, they have made outstanding contributions to the development of the disciplines of environmental toxicology and chemistry. Our study systematically summarizes the toxicological effects of MNPs size, concentration, and exposure duration on mice/rat models. There are a few studies on the toxicological effects of MNPs on rats, and the endpoints of rats were all <3 studies; therefore, the meta-analysis only analyzed those four studies on mice. The sensitivities of different strains of mice to MNPs exhibit significant differences, which are also one of the sources of heterogeneity.

All the included literature were divided into seven categories and 24 subcategories according to the different physiological functions and toxicity endpoints affected by MNPs, several of which, such as the nervous, reproduction, and gastrointestinal and metabolic systems, are worthy of attention. MNPs can be absorbed into the bloodstream through the gastrointestinal tract and deposited in the brains of mice, which causes neurotoxicity and attracts our attention. Our findings are consistent with the results of previous reviews (75). The toxicological effect of MNPs on the nervous system of mice was reflected in the fact that it can lead to brain PD-like neurodegeneration and behavioral and motor disorders and served as a potential risk factor for autism spectrum disorder (ASD). Studies have shown that toxicological effects were induced by MNPs to the mouse brain, which might lead to PD-like neurodegeneration mainly through the disturbance in energy metabolism in brain cells (59). MNPs can be transmitted at different nutritional levels through the food chain and eventually enter the body of mice, causing behavioral disorders (76). Another study examined the behavioral changes in mice of different ages through maternal exposure to MNPs, ultimately identifying MNPs as a potential contributor to ASD (77). Another study showed that the effect of PS-NPs on neurobehavior in rats was not statistically significant due to the limitations of small sample sizes and study design (78). There is an urgent need for more studies to further clarify the neurotoxic hazards and risks of MNP exposure.

Micro/nano-plastics have a significant impact on the reproductive function of mice. Reproductive toxicity mainly includes sperm deformities, testicular inflammation, and decreased testosterone levels in male mice (79) and ovarian inflammation and the quality reduction of oocytes in female mice (61). Several studies have further explored the transmission effect of exposing MNPs to the next generation (80–82). Moreover, they found that maternal exposure to MNPs in mice showed effects on the growth of offspring. Our results displayed that the male mice were more sensitive to the reproductive toxicity of MNPs. The toxicity of MNPs to the reproductive system of rats has mainly resulted in the deficiency of ovarian function in female mice (83). PS-MPs could cause apoptosis of ovarian granulosa cells through the NLRP3/Caspase-1 signaling pathway, and its mechanism might be related to oxidative stress (84). To date, most of the toxicological studies of MNPs on the reproductive system of mice have focused only on male mice; therefore, studies on the effects of the female reproductive system need to be carried out, and the lack of them has raised the issue of the need for a more reliable and extensive analysis.

Direct exposure of MNPs to the gastrointestinal system could result in an injury to the digestive tract tissue (33). Moreover, MNPs can accumulate in the intestinal tissue of mice, reduce intestinal mucus secretion, damage the intestinal barrier function, and change the diversity of intestinal flora (65). Therefore, it is necessary to explore the correlation between MNPs and the intestinal microflora, including the signaling pathways.

Exposure to MNPs poses a threat to the metabolic system of mouse/rat models. Among them, the biological endpoint of oxidative stress was the most concerning factor. Oxidative stress-related biological endpoints were assessed using relevant biomarkers, and the related signaling pathways were also identified (34). It also demonstrated that long-term MP exposure was a key risk factor that elicited oxidative stress in mice. MNPs could mediate the MAPK–Nrf2 signaling pathway by inducing oxidative stress, leading to the disruption of the blood–testis barrier in rats; thereby affecting the reproductive performance of rats (51). Another study found that MNPs could induce oxidative stress by activating the fibrosis-related Wnt/β-catenin signaling pathway to induce apoptosis in rat cardiomyocytes (71). MNPs induce reproductive toxicity, behavioral changes, and other adverse consequences through the oxidative stress cascade reaction and inflammatory reaction. At the same time, MNPs caused a disturbance in blood glucose and lipid metabolism in mice/rat models. The liver triglyceride and total cholesterol levels became reduced in mice treated with high concentrations of MPs, while no significant effects were observed in the low-concentration group (52). The disorder effect of blood glucose metabolism happened during >3 Class MPs exposure time. Normalized experiments for exploration of the exposure dose–/time–response relationships of MNPs should be established.

Micro/nano-plastics can enter the blood circulation of mice/rat models and become deposited in key tissues and target organs. During the deposition processes, they cause immune system disorders, blood toxicity, and organ damage, such as to the liver, kidneys, and lungs (36, 69, 85). It was found that MNPs could reduce the peripheral blood cell count by inhibiting the differentiation in bone marrow hematopoietic stem cells (67). MNPs increased the infiltration of natural killer cells and stimulation of cells into hepatocytes, and the expression of the inflammatory factor was upregulated. Another study found that MNPs cause oxidative stress, inflammation, and autophagy responses in renal cells; meanwhile, MNPs can accumulate in HK-2 cells and mouse kidneys (86). We found that 65.22% of the endpoints of visceral organ injury were significantly affected, which contributes to a few studies on this category with only 69 endpoints. Further studies are required to better understand the impact of MNPs on visceral organ injury.

Polystyrene and polyethylene are the most commonly used types and ingredients of MNP polymers in current experiments with mice and rat models. We explored the effects of different types of MPs on blood glucose and lipid metabolism in mice, and we observed that PS tended to have a more obvious effect on substance metabolism in mice than PE. It might be that there are fewer studies on the use of PE. It also found that functional amino acid- and carboxyl-group modified PS was more toxic than the original one, and the toxicity of PS-NH_2_ was stronger than that of PS-COOH (65). The difference in toxicity might be due to the different surface charges in MNPs, and the charged PS-NH_2_ and PS-COOH are more easily transported through the cellular membrane. Only one report tested the toxicity of rats by intragastric administration by using PP without observation of adverse reactions (87). More polymer types of MNPs should be explored and a comparison of their toxic effects should be made. In terms of human health, the most common polymers of MNPs that we are exposed to in daily life include not only PS and PE but also polyethylene terephthalate (9, 22). However, we are yet to find any animal tests and epidemiology reports on the effects of PS or PE on human health.

Size is considered to be a key factor affecting the toxicity of MNPs. Particles between 10 and 100 nm in size are more beneficial to pinocytosis and those between 4 and 10 nm in size can go through the membrane bilayer by direct penetration, which might be due to the large specific surface area of these small particles and induce a more efficient interaction with cells (88). NPs of 100 nm in size can penetrate the cell membrane more easily than MPs with larger sizes and accumulate in tissues and cells (74). Moreover, the surface mass ratio of NPs is larger than that of MPs, which enables NPs to more readily penetrate the lipid membrane directly (34). It was similar to our results, suggesting that NPs could damage the nervous and reproductive systems in mice easily (Figure 3). Similarly, the surface charge potential and the mass surface ratio of NPs make it easier to absorb free radicals. It has been reported that most MNPs with particle diameters smaller than 2.5–5 μm can enter the systemic circulation, thereby reaching multiple organs of the body and causing physiological damage (15).

The sizes of MPs detected in human feces, sputum, and breast milk were 20–800, 20–500, and 2–12 μm, respectively (10, 24, 89). Owing to the limitations in standard detection technology, it is currently difficult to detect all kinds of nanoscaled plastic particles in human samples. A comprehensive analysis of different biological tissue levels should be performed to discover the potential mechanism of NPs on organisms.

Exposure assessment should focus on the accumulation of MNPs into various tissues and organs and their ability to cross potential biological barriers to better detect the damage to health caused by MNPs (75). It has been reported that 488-nm PS–NPs can be deposited in the liver, intestine, and liver tissues of mice (90). Experiments performed on inhalation toxicity showed that 1-5 μm MPs could induce the infiltration of inflammatory cells in the lung tissue and the accumulation of bronchoalveolar macrophages (69). PS–NPs (80 nm) were found in multiple organs including the liver, kidney, and brain of the mice and caused damage to internal organs after their oral administration to mice (46). The size of MNPs is an important factor. A large particle is easy to accumulate in the digestive system and the respiratory system, whereas a small particle can enter the vital organs, including the brain, placenta, kidney, liver, and other organs, through biological barriers. The health risks of long-term exposure cannot be ignored.

The toxicity of MNPs also depends on the exposure concentration and dose. A study explored the toxicological effects of MNPs on mice through acute and repeated exposures, demonstrating that the lethal dose of MNPs to rats was higher than 2,000 mg/kg (50). Five groups of different concentrations ranging from 0 to 2,000 μgL^−1^ were set up to explore the effects of MNPs on the reproductive system of rats in another study (91). It was found that MNPs caused oxidative stress, reducing the number of germ cells in rats and causing potential damage to the testis of rats in a dose-dependent manner. There is still a lack of data to explore the MNP concentration range of the effects on the reproductive system of mice/rat models, which is partly because of the different exposure and concentration calculation methods. Therefore, standardized exposure methods should be established for a better comparison between different studies.

Exposure time is also one of the important factors affecting the toxicity of MNPs. Reproductive toxicity depends on the concentration and exposure time, and a higher concentration and longer exposure time can lead to stronger toxicity. Studies using longer exposure times tend to adopt lower exposure concentrations, which might be due to the fact that only a few biological endpoints are affected by a longer exposure time. A previous study found that MPs affected the count and quality of sperm in mice by exposing them to 100 μg/L/day MPs for 180 days (35), which was much lower than the concentration of Lee's report, i.e., 2,000 mg/kg (50). The histopathologic evaluation revealed significant foreign body inflammation in the lung tissue in the 28-day repeated oral group (50). Therefore, a long-term repeated exposure to MNPs can lead to toxicological effects on mice/rat models.

The concentration and duration of exposure of mice to MNPs based on laboratory conditions are different from those of humans exposed to MNPs under the indicated conditions. A previous study suggested that a study that uses low-level prolonged exposures should be designed, which can get closer to real human exposures (92). Another study reported that commonly used plastic consumer goods, such as disposable food-grade nylon bags and hot low-density PE drink-cup liners, release NPs more than 10^12^ L^−1^ (93). On average, humans might ingest 0.1 to 5 g of MNPs per week through various routes of exposure (94). The concentrations and exposure times still need to be closer to the real levels. At present, the total daily intake of MNPs is mainly simulated by calculating the intake of water, food and inhaled air (16, 94). However, this method still has a few limitations because the MNPs released by the mask were not included in this model due to the COVID-19 pandemic.

Micro/nano-plastics (MNPs) can enter the organism *in vivo* through the digestive tract, the respiratory tract, and skin contact (16). Owing to the technical limitations and without standard methods used in the extraction, identification, and quantification of MNPs, it is hard to obtain the real concentration of MNPs in the environment and the exact exposure level of organisms. It has been reported that the concentration of MPs in the fecal samples of eight humans is 2 items/g faces (8). Another study extracted MPs from 50 healthy people and 52 patients with inflammatory bowel disease (IBD), and it was found that the average concentrations of MPs were 28 and 41.8 items/g faces, respectively (10). MNPs can be released into the air in all kinds of household products (11). In a study elaborating on the collection of sputum samples drawn from 22 patients with respiratory diseases, MPs were prevalent in all these sputum samples (89). A simulation of the ingestion of MNPs through respiration exhibited that an adult male could inhale 272 MPs/day (95). Plastic beads are added to many personal care products (96); therefore, the long-term use of such personal care products will lead to the exposure of MNPs in the human body.

At present, there is no clear evidence to support the impact of microplastics on human health. The survey found a positive correlation between fecal MPs and IBD status, suggesting that MP exposure may be related to the disease process or that IBD exacerbates the retention of MPs (10). The point is that more epidemiological studies are needed to fill in the gaps. Judging from the current detection of MNPs in human samples, the health risks of long-term exposure to humans are not optimistic.

The results of the present study also have some limitations. Ingestion and inhalation are the two main exposure patterns of human exposure to MNPs. Animal models ingest MNPs by gavage, and most of the doses of exposure were much higher than those ingested by human beings. In total, 5.1 × 10^3^ MNPs particles/day can be ingested by an adult from salt and 4.1 × 10^4^ items from drinking water, and the average annual inhalation intake of MNPs can reach 0.9 × 10^4^ to 7.9 × 10^4^ (15). The comparison between different exposure pathways and species is limited, and appropriate methods should be found for the actual amount of human MP exposure. From a medical ethics point of view, it is not possible to apply to or impose a chemical contaminant on a person to determine its actual dose. However, there is no clear method to assess the actual exposure of MPs.

## 5. Conclusion

The present study has systematically summarized the toxic effects of MNPs on mouse/rat models. The findings of the present study demonstrate that 52.78% of biological endpoints were found to be significantly affected by exposure to MNPs. These findings also provide insights into and directions for exploring the evidence and mechanisms of MNP effects on human health. However, further studies are required on the pathological mechanisms at the molecular level and the long-term and chronic effects of tissue accumulation. This review also aimed to guide future studies for establishing a standard MNP exposure model in rats and mice, and a further investigation of MNP exposure under comprehensive and real-world conditions, the potential toxic mechanisms, and the health effects.

## Data availability statement

The original contributions presented in the study are included in the article/Supplementary material, further inquiries can be directed to the corresponding authors.

## Author contributions

ZQ and NL were involved in the administrative support, conception, and design of the manuscript, data interpretation, reviewing, and revising the manuscript. WL was involved in the conception and design of the manuscript, collection and assembly of the data, data analysis, and drafting of the initial manuscript. BZ, QY, and XF were involved in the collection and assembly of data. TS, PG, and PaW were involved in drafting the initial manuscript. YB, BL, and PeW were involved in the critical review of the manuscript for important intellectual content. RL was involved in data analysis. All authors contributed to the article and approved the submitted version.
