# Supplementary material for: Toxicological effects of micro/nano-plastics on mouse/rat models: a systematic review and meta-analysis
Source: Front Public Health. 2023 May 18;11:1103289. doi: 10.3389/fpubh.2023.1103289 (PMC10233117; doi:10.3389/fpubh.2023.1103289)
Supplement: Supplementary file 1 [file Data_Sheet_1.docx]

**Supplementary Information**

**Toxicological effects of micro/nano-plastics on mouse/rat models: a systematic review and meta-analysis**

Weijia Liu^1, 2^, Bowen Zhang ^1^, Qianqian Yao ^1^, Xihua Feng, ^1, 3^, Tianling Shen, ^1, 3^, Peisen Guo^1, 3^, Panpan Wang^1, 3^, Yitong Bai ^1, 2^, Bo Li ^1^, Peixi Wang ^1^, Ruiling Li ^1^, Zhi Qu ^1 *^
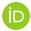
, Nan Liu ^1, 2, 3*^
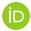


^1^ *Institute of Environment and Health, South China Hospital, Shenzhen University, Shenzhen, 518116, P. R. China*

^2^ *Institute of Chronic Disease Risks Assessment, School of Nursing and Health, Henan University, Kaifeng, 475004, P. R. China*

^3^ *College of Public Health, Zhengzhou University, Zhengzhou, 540001, P. R. China*

***Corresponding authors:**

*Zhi Qu*

E-mail: 10210056@henu.edu.cn

ORCID: https://orcid.org/0000-0001-9898-4746

*Nan Liu*

E-mail: [13688869875@163.com](mailto:13688869875@163.com)

ORCID: <https://orcid.org/0000-0002-8895-3169>

**Summary: Supporting information contains 8 figures, and 1 table**

**Contents**

1. **Fig. S1** Funnel plots to check potential for publication bias of glucose metabolism in mice

2. **Fig. S2** Funnel plots to check potential for publication bias of reproduction in mice

3. **Fig. S3** Funnel plots to check potential for publication bias of lipid metabolism in mice

4. **Fig. S4** Funnel plots to check potential for publication bias of oxidative stress in mice

5. **Fig. S5** Sensitivity analyses of the effect size of glucose metabolism in mice

6. **Fig. S6** Sensitivity analyses of the effect size of mice reproduction

7. **Fig. S7** Sensitivity analyses of the effect size of mice lipid metabolism

8. **Fig. S8** Sensitivity analyses of the effect size of mice oxidative stress

9. **Table S1** Evaluation of risk of bias in studies with animal models according to the SYRCLE tool


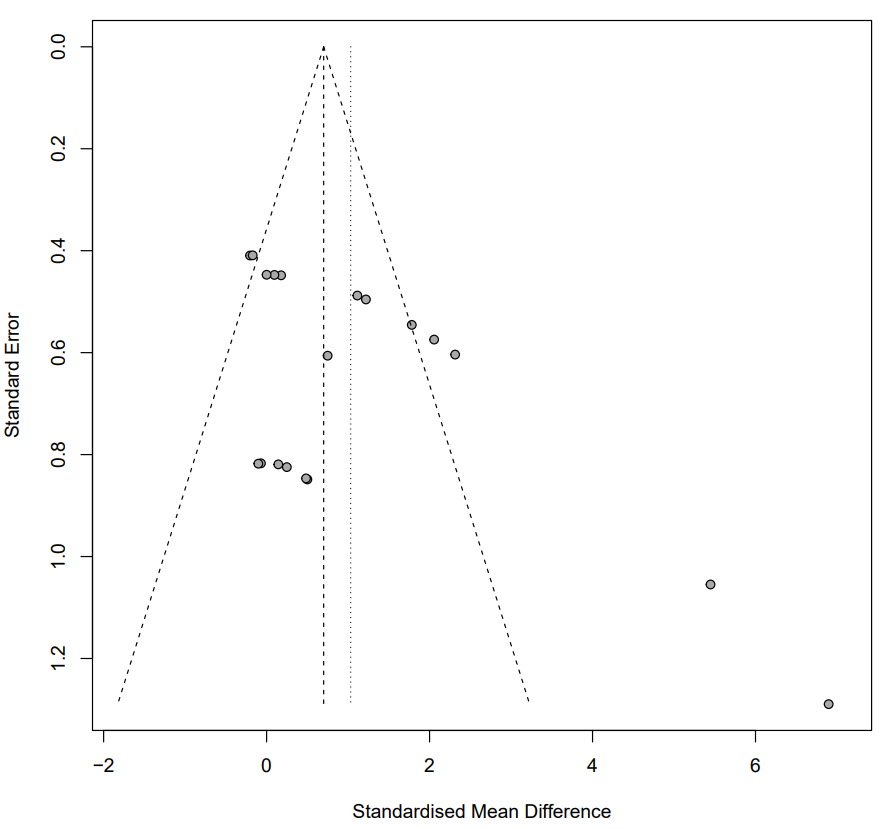


**Fig. S1** Funnel plots to check potential for publication bias of glucose metabolism in mice


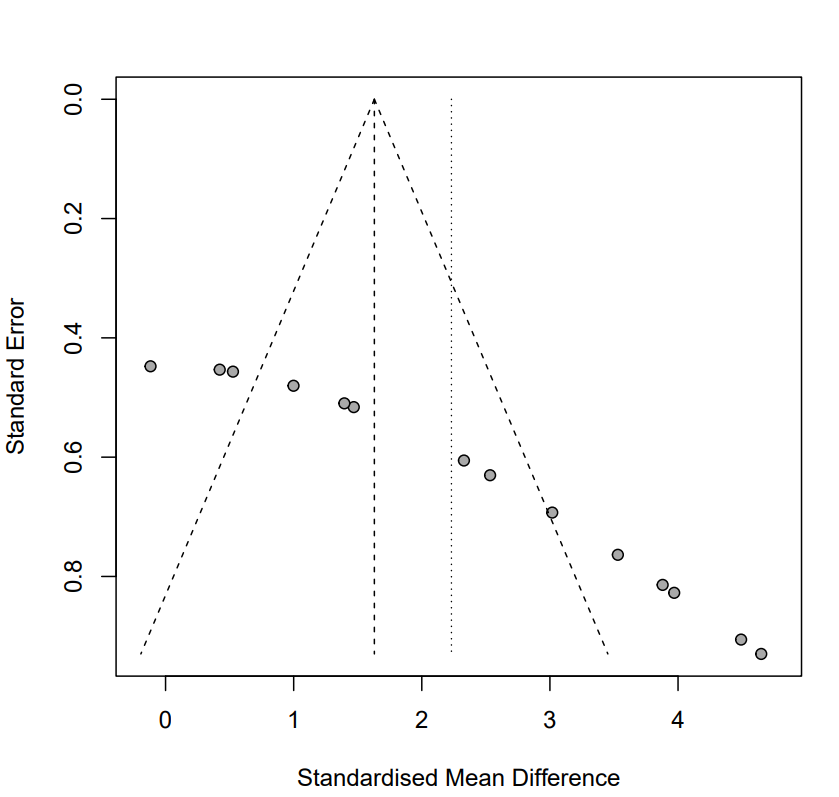


**Fig. S2** Funnel plots to check potential for publication bias of reproduction in mice


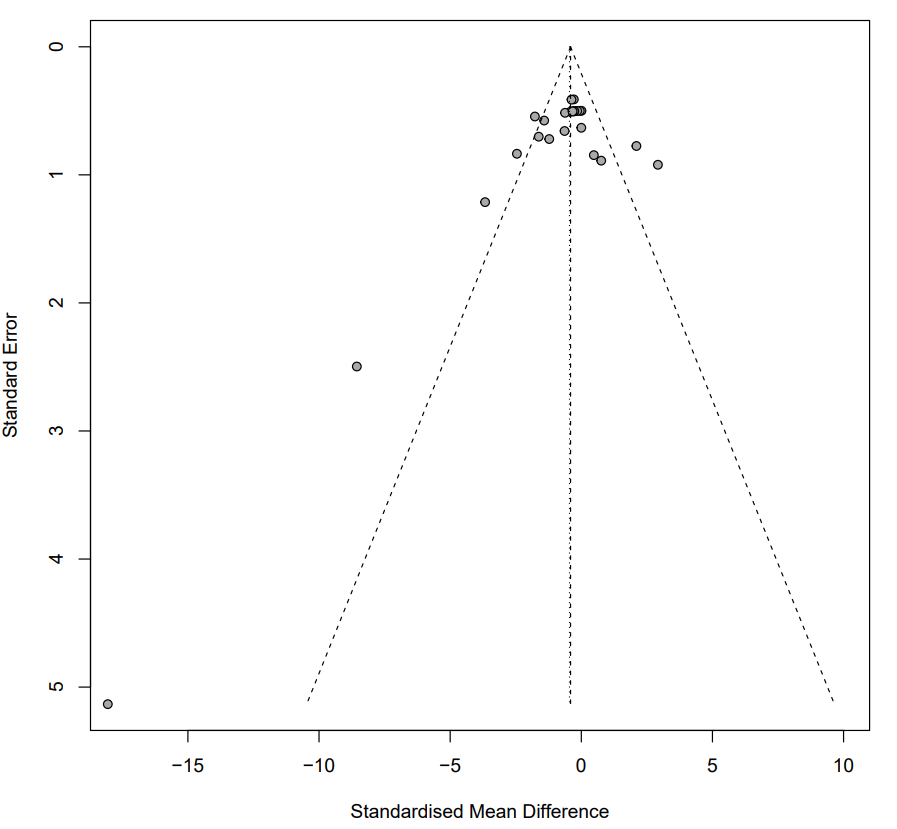


**Fig. S3** Funnel plots to check potential for publication bias of lipid metabolism in mice


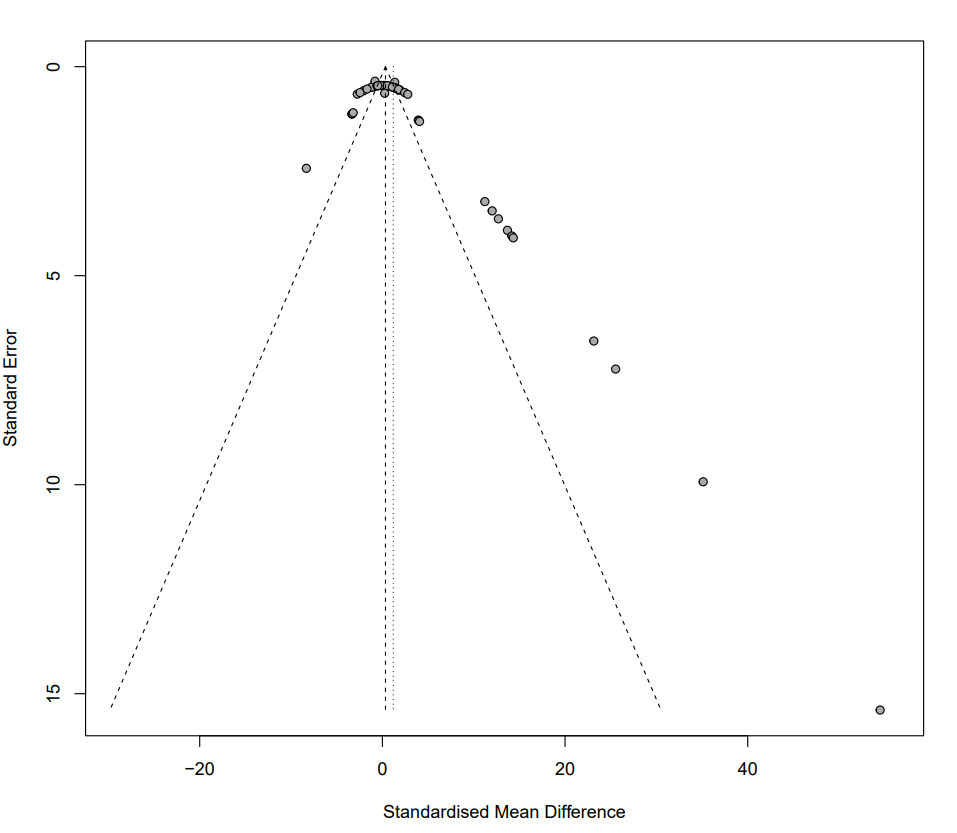


**Fig. S4** Funnel plots to check potential for publication bias of oxidative stress in mice

**
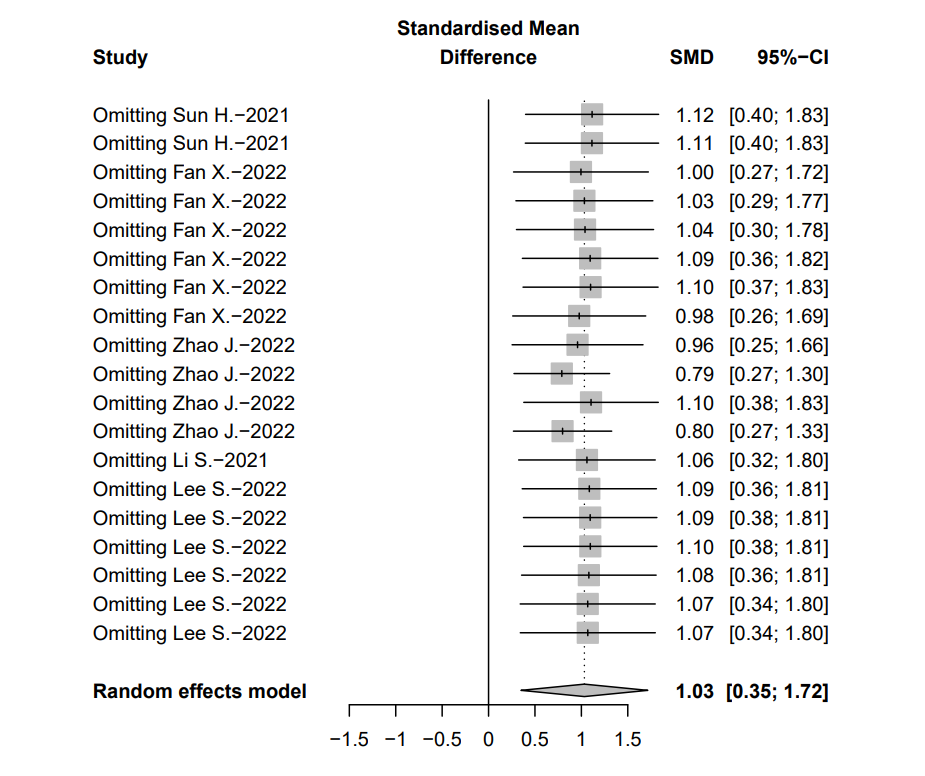
**

**Fig. S5** Sensitivity analyses of the effect size of glucose metabolism in mice

**
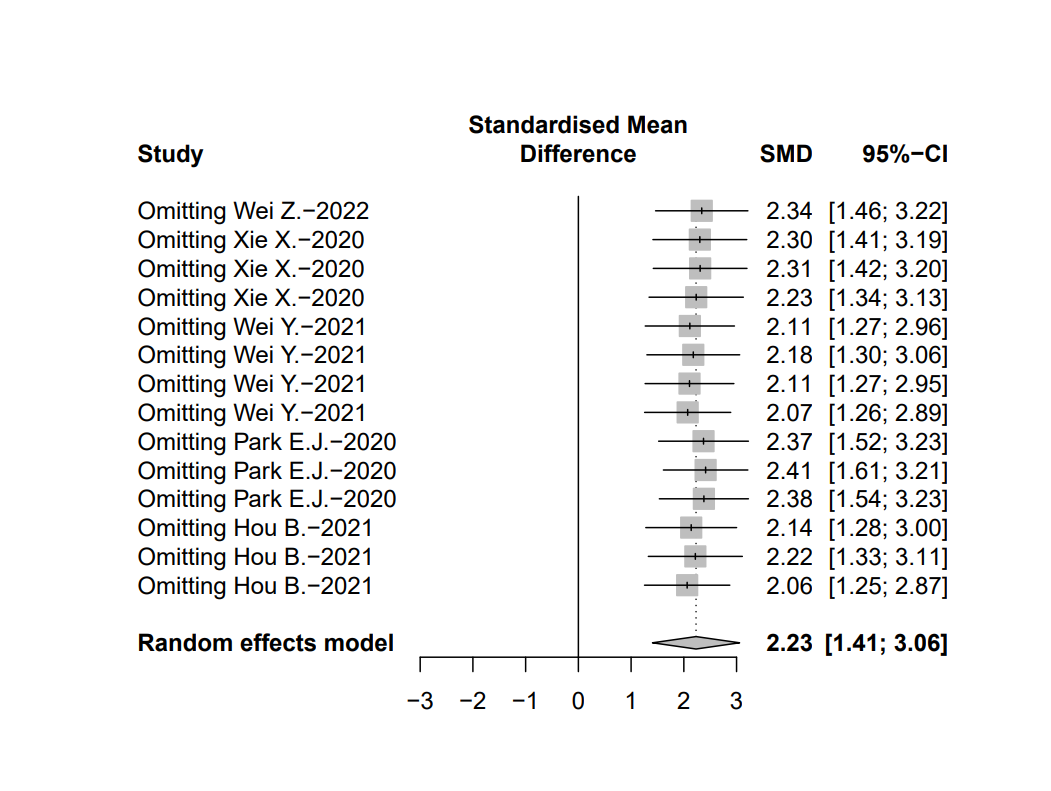
**

**Fig. S6** Sensitivity analyses of the effect size of mice reproduction


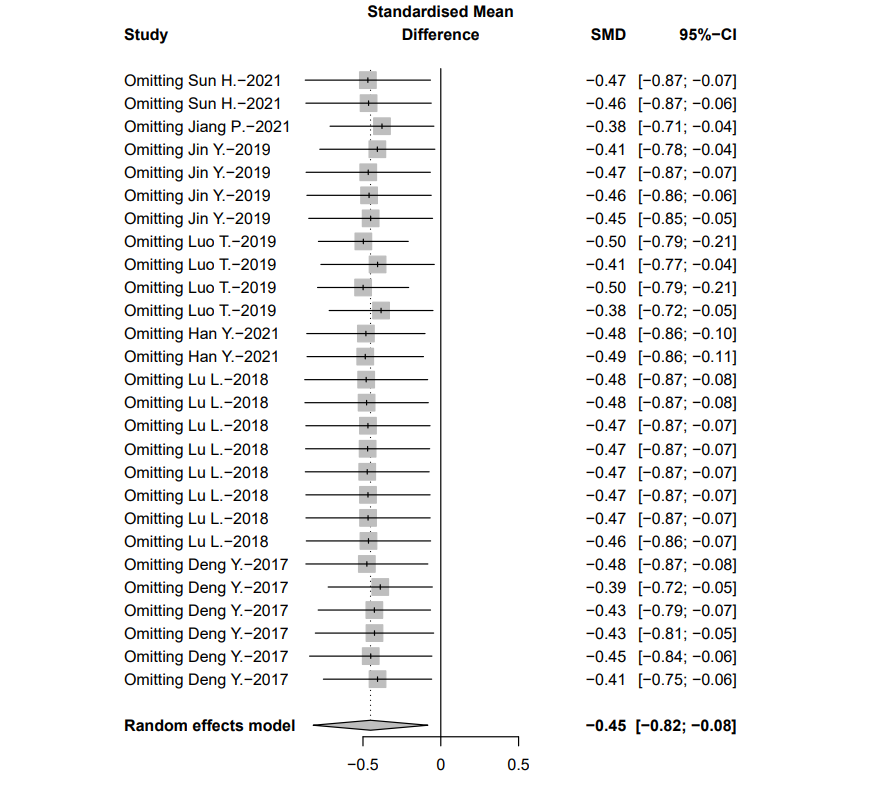


**Fig. S7** Sensitivity analyses of the effect size of mice lipid metabolism


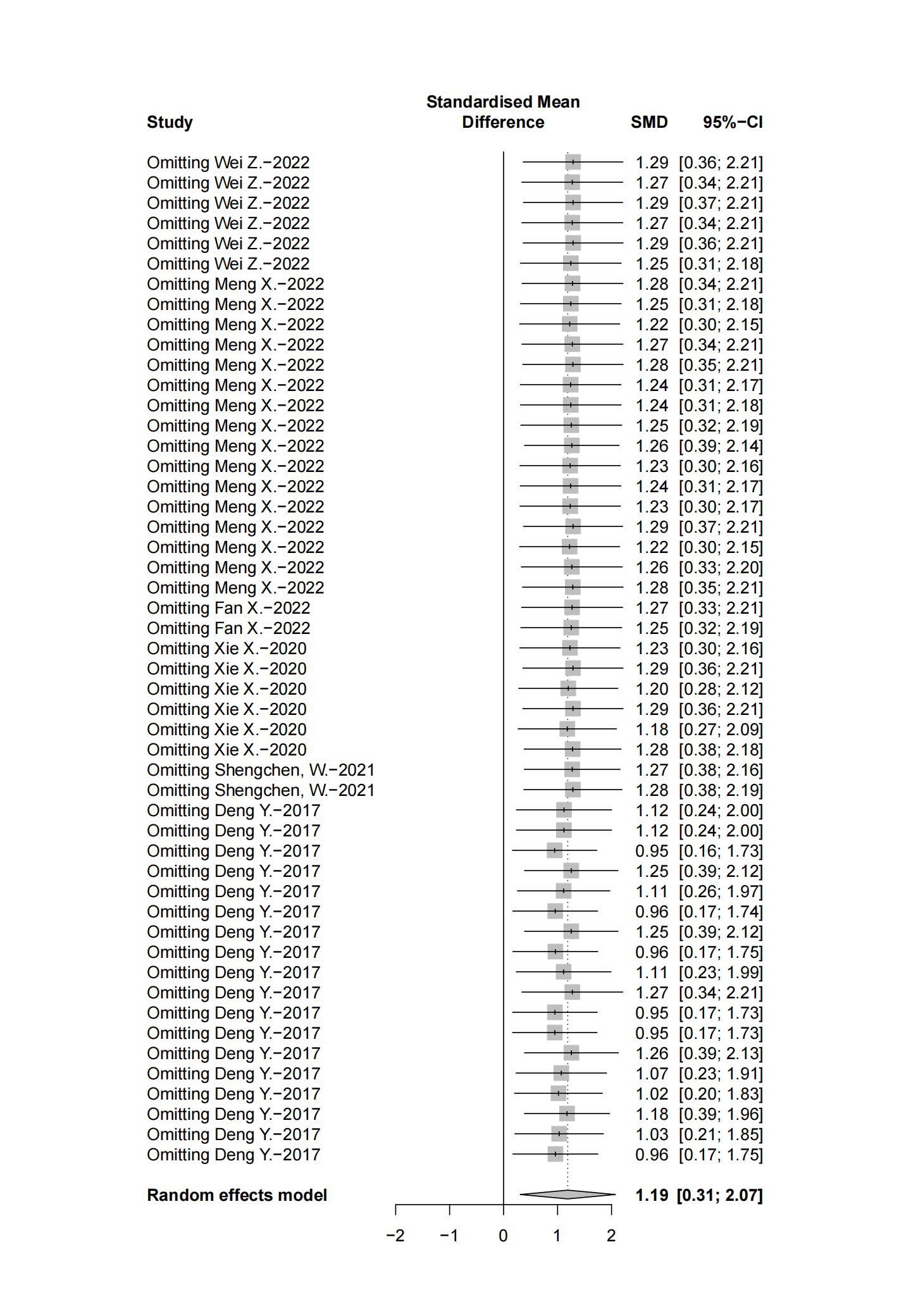


**Fig. S8** Sensitivity analyses of the effect size of mice oxidative stress

**Table S1**. Evaluation of risk of bias in studies with animal models according to the SYRCLE tool

| **First Author** | **Q1** | | **Q2** | | **Q3** | | **Q4** | | **Q5** | | **Q6** | | **Q7** | | **Q8** | | **Q9** | | **Q10** | | **References** | |  |
| --- | --- | --- | --- | --- | --- | --- | --- | --- | --- | --- | --- | --- | --- | --- | --- | --- | --- | --- | --- | --- | --- | --- | --- |
| Mu Y. | Y | | Y | | ? | | ? | | ? | | Y | | ? | | Y | | Y | | Y | | (Mu et al., 2022) | |  |
| Kim J. | Y | | Y | | ? | | ? | | ? | | ? | | ? | | Y | | Y | | Y | | (Kim et al., 2021) | |  |
| Wang Y. | Y | | Y | | ? | | ? | | ? | | ? | | ? | | Y | | Y | | Y | | (Wang et al., 2022) | |  |
| Liang B. | Y | | Y | | ? | | ? | | N | | ? | | ? | | Y | | Y | | Y | | (Liang et al., 2022) | |  |
| Jin H. | Y | | ? | | N | | ? | | ? | | ? | | ? | | Y | | Y | | Y | | (Jin et al., 2022) | |  |
| Wei Z. | Y | | ? | | ? | | ? | | N | | Y | | ? | | Y | | Y | | Y | | (Wei et al., 2022) | |  |
| Lu K. | ? | | ? | | ? | | ? | | ? | | ? | | ? | | Y | | Y | | Y | | (Lu et al., 2021) | |  |
| Ijaz, M. U | Y | | Y | | ? | | N | | ? | | ? | | ? | | Y | | Y | | Y | | (Ijaz et al., 2021) | |  |
| Sun H. | Y | | ? | | ? | | ? | | ? | | ? | | ? | | Y | | Y | | Y | | (Sun et al., 2021) | |  |
| Jiang P. | Y | | ? | | N | | ? | | ? | | ? | | ? | | Y | | Y | | Y | | (Jiang et al., 2021) | |  |
| Kang K. | Y | | Y | | ? | | ? | | ? | | Y | | ? | | Y | | Y | | ? | | (Kang et al., 2019) | |  |
| Meng X. | Y | | Y | | ? | | ? | | ? | | Y | | ? | | Y | | Y | | Y | | (Meng et al., 2022) | |  |
| Fan X. | Y | | Y | | ? | | ? | | ? | | ? | | ? | | Y | | Y | | Y | | (Fan et al., 2022) | |  |
| Li H. | Y | | Y | | ? | | ? | | ? | | Y | | N | | Y | | Y | | Y | | (Li et al., 2021) | |  |
| Haddadi A. | Y | | Y | | ? | | ? | | ? | | ? | | ? | | Y | | Y | | Y | | (Haddadi et al., 2022) | |  |
| Xie X. | Y | | ? | | ? | | ? | | ? | | ? | | ? | | Y | | Y | | Y | | (Xie et al., 2020) | |  |
| Lee C.W. | Y | | Y | | ? | | ? | | ? | | ? | | ? | | Y | | Y | | Y | | (Lee et al., 2022) | |  |
| Jin Y. | Y | | Y | | ? | | ? | | N | | ? | | ? | | Y | | Y | | Y | | (Jin et al., 2019) | |  |
| Choi Y.J. | Y | | ? | | ? | | ? | | N | | ? | | ? | | Y | | Y | | Y | | (Choi et al., 2021) | |  |
| Choi Y.J | Y | | ? | | ? | | ? | | ? | | ? | | ? | | Y | | Y | | Y | | (Choi et al., 2021) | |  |
| Marlene S. | Y | | Y | | ? | | ? | | ? | | ? | | ? | | Y | | Y | | Y | | (Marlene et al., 2022) | |  |
| Li X. | Y | | ? | | ? | | ? | | N | | ? | | ? | | Y | | Y | | Y | | (Li et al., 2022) | |  |
| Luo T. | Y | | ? | | N | | ? | | ? | | ? | | ? | | Y | | Y | | Y | | (Luo et al., 2019) | |  |
| Jeong B. | Y | | ? | | ? | | ? | | ? | | ? | | ? | | Y | | Y | | Y | | (Jeong et al., 2022) | |  |
| Huang T. | Y | | ? | | ? | | N | | ? | | ? | | ? | | Y | | Y | | Y | | (Huang et al., 2022) | |  |
| Luo T. | Y | | Y | | ? | | ? | | ? | | ? | | ? | | Y | | Y | | Y | | (Luo et al., 2019) | |  |
| Kwon W. | Y | | Y | | ? | | ? | | ? | | ? | | ? | | Y | | Y | | Y | | (Kwon et al., 2022) | |  |
| Rawle D. | | Y | | Y | | ? | | N | | ? | | ? | | ? | | Y | | Y | | Y | | (Rawle et al., 2022) | |
| da Costa. | | Y | | Y | | N | | ? | | ? | | ? | | ? | | Y | | Y | | Y | | (da Costa et al.,2021) | |
| Babaei A. | | Y | | Y | | ? | | ? | | ? | | ? | | N | | Y | | Y | | Y | | (Babaei et al., 2021) | |
| Rafiee M. | | Y | | Y | | ? | | ? | | ? | | ? | | ? | | Y | | Y | | Y | | (Rafiee et al., 2018) | |
| Han Y. | | Y | | Y | | ? | | ? | | ? | | ? | | ? | | Y | | Y | | Y | | (Han et al., 2021) | |
| Choi Y.J. | | Y | | Y | | ? | | ? | | ? | | ? | | ? | | Y | | Y | | Y | | (Choi et al., 2021) | |
| Qiao J. | | Y | | Y | | ? | | ? | | ? | | ? | | ? | | Y | | Y | | Y | | (Qiao et al., 2021) | |
| Li B. | | Y | | ? | | N | | ? | | ? | | ? | | ? | | Y | | Y | | Y | | (Li et al., 2020) | |
| Zhao J. | | Y | | Y | | ? | | ? | | ? | | ? | | ? | | Y | | Y | | Y | | (Zhao et al., 2022) | |
| Jing J. | | Y | | ? | | ? | | ? | | ? | | ? | | N | | Y | | Y | | Y | | (Jing et al., 2022) | |
| Li Z. | | Y | | Y | | ? | | ? | | ? | | N | | ? | | Y | | Y | | Y | | (Li et al., 2020) | |
| Wei Y. | | Y | | ? | | ? | | ? | | ? | | ? | | ? | | Y | | Y | | Y | | (Wei et al., 2021) | |
| Hu J. | | Y | | Y | | ? | | ? | | N | | ? | | ? | | Y | | Y | | Y | | (Hu et al., 2021) | |
| Li S. | | Y | | Y | | ? | | ? | | ? | | ? | | ? | | Y | | Y | | Y | | (Li et al., 2021) | |
| Lu L. | Y | | Y | | ? | | ? | | ? | | ? | | ? | | Y | | Y | | Y | | (Lu et al., 2018) | |  |
| Liu Z. | Y | | Y | | ? | | N | | ? | | ? | | ? | | Y | | Y | | Y | | (Liu et al., 2022) | |  |
| Jin H. | Y | | Y | | ? | | ? | | ? | | ? | | ? | | Y | | Y | | Y | | (Jin et al., 2021) | |  |
| Hou J. | Y | | Y | | ? | | ? | | ? | | ? | | ? | | Y | | Y | | Y | | (Hou et al., 2021) | |  |
| Li S. | Y | | Y | | ? | | ? | | ? | | ? | | ? | | Y | | Y | | Y | | (Li et al., 2021) | |  |
| Wang S. | Y | | Y | | ? | | ? | | ? | | ? | | ? | | Y | | Y | | Y | | (Wang et al., 2021) | |  |
| Zaheer J. | Y | | Y | | ? | | ? | | ? | | N | | ? | | Y | | Y | | Y | | (Zaheer et al., 2022) | |  |
| Sun R. | Y | | ? | | ? | | N | | ? | | ? | | ? | | Y | | Y | | Y | | (Sun et al., 2021) | |  |
| Zheng H. | Y | | Y | | ? | | ? | | ? | | ? | | N | | Y | | Y | | Y | | (Zheng et al., 2021) | |  |
| Zhao L. | Y | | ? | | ? | | ? | | ? | | ? | | ? | | Y | | Y | | Y | | (Zhao et al., 2021) | |  |
| Park E.J. | Y | | Y | | ? | | ? | | ? | | ? | | ? | | Y | | Y | | Y | | (Park et al., 2020) | |  |
| Hou B. | Y | | Y | | ? | | ? | | ? | | ? | | ? | | Y | | Y | | Y | | (Hou et al., 2021) | |  |
| Xiao J. | Y | | Y | | ? | | ? | | ? | | N | | ? | | Y | | Y | | Y | | (Xiao et al., 2022) | |  |
| Ahmed Y. H. | Y | | Y | | ? | | ? | | ? | | ? | | ? | | Y | | Y | | Y | | (Ahmed et al., 2022) | |  |
| Xu D. | Y | | Y | | ? | | ? | | N | | ? | | ? | | Y | | Y | | Y | | (Xu et al., 2021) | |  |
| Amereh, F. | Y | | Y | | ? | | ? | | ? | | ? | | ? | | Y | | Y | | Y | | (Amereh et al., 2020) | |  |
| Tian K. | Y | | Y | | ? | | ? | | ? | | ? | | ? | | Y | | Y | | Y | | (Tian et al., 2020) | |  |
| Wei J. | Y | | Y | | ? | | ? | | ? | | ? | | ? | | Y | | Y | | Y | | (Wei et al., 2021) | |  |
| Wang Y. | Y | | ? | | ? | | ? | | ? | | ? | | ? | | Y | | Y | | Y | | (Wang et al., 2021) | |  |
| Amereh, F. | Y | | Y | | ? | | ? | | ? | | ? | | ? | | Y | | Y | | Y | | (Amereh et al., 2019) | |  |
| Deng Y. | Y | | Y | | ? | | ? | | N | | ? | | ? | | Y | | Y | | Y | | (Deng et al., 2017) | |  |
| Lee S. | Y | | ? | | N | | ? | | ? | | ? | | ? | | Y | | Y | | Y | | (Lee et al., 2022) | |  |
| Liang B. | Y | | ? | | ? | | ? | | N | | ? | | ? | | Y | | Y | | Y | | (Liang et al., 2021) | |  |
| Stock V. | Y | | ? | | ? | | ? | | ? | | ? | | ? | | Y | | Y | | Y | | (Stock et al., 2019) | |  |

Q1: Was the allocation sequence adequately generated and applied?; Q2: Were the groups similar at baseline?; Q3: Was the allocation to the different groups adequately concealed during?; Q4: Were the animals randomly housed during the experiment?; Q5: Were the caregivers and/or investigators blinded from knowledge which intervention each animal received during the experiment?; Q6: Were animals selected at random for outcome assessment?; Q7: Was the outcome assessor blinded?; Q8: Were incomplete outcome data adequately addressed?; Q9: Are reports of the study free of selective outcome reporting?; Q10: Was the study apparently free of other problems that could result in high risk of bias?

Y: low risk of bias; N: high risk of bias; ?: unclear risk of bias. It is not recommend calculating a summary score for each individual study when using this toll.

**References**

Ahmed YH, El-Naggar ME, Rashad MM, A MY, Galal MK, Bashir DW (2022) Screening for polystyrene nanoparticle toxicity on kidneys of adult male albino rats using histopathological, biochemical, and molecular examination results. Cell Tissue Res 388:149-165. https://doi.org/10.1007/s00441-022-03581-5

Amereh F, Babaei M, Eslami A, Fazelipour S, Rafiee M (2020) The emerging risk of exposure to nano(micro)plastics on endocrine disturbance and reproductive toxicity: From a hypothetical scenario to a global public health challenge. Environ Pollut 261:114158. https://doi.org/10.1016/j.envpol.2020.114158

Amereh F, Eslami A, Fazelipour S, Rafiee M, Zibaii MI, Babaei M (2019) Thyroid endocrine status and biochemical stress responses in adult male Wistar rats chronically exposed to pristine polystyrene nanoplastics. Toxicol Res (Camb) 8:953-963. https://doi.org/10.1039/c9tx00147f

Babaei AA, Rafiee M, Khodagholi F, Ahmadpour E, Amereh F (2021) Nanoplastics-induced oxidative stress, antioxidant defense, and physiological response in exposed Wistar albino rats. Environ Sci Pollut Res 29:11332-11344. https://doi.org/10.1007/s11356-021-15920-0

Choi YJ, Kim JE, Lee SJ, Gong JE, Jin YJ, Seo S, Lee JH, Hwang DY (2021a) Inflammatory response in the mid colon of ICR mice treated with polystyrene microplastics for two weeks. Lab Anim Res 37:31. https://doi.org/10.1186/s42826-021-00109-w

Choi YJ, Park JW, Kim JE, Lee SJ, Gong JE, Jung YS, Seo S, Hwang DY (2021b) Novel Characterization of Constipation Phenotypes in ICR Mice Orally Administrated with Polystyrene Microplastics. Int J Mol Sci 22 https://doi.org/10.3390/ijms22115845

Choi YJ, Park JW, Lim Y, Seo S, Hwang DY (2021c) In vivo impact assessment of orally administered polystyrene nanoplastics: biodistribution, toxicity, and inflammatory response in mice. Nanotoxicology 15:1180-1198. https://doi.org/10.1080/17435390.2021.1996650

da Costa Araujo AP, Malafaia G (2021) Microplastic ingestion induces behavioral disorders in mice: A preliminary study on the trophic transfer effects via tadpoles and fish. J Hazard Mater 401:123263. https://doi.org/10.1016/j.jhazmat.2020.123263

Deng Y, Zhang Y, Lemos B, Ren H (2017) Tissue accumulation of microplastics in mice and biomarker responses suggest widespread health risks of exposure. Sci Rep 7:46687. https://doi.org/10.1038/srep46687

Fan X, Wei X, Hu H, Zhang B, Yang D, Du H, Zhu R, Sun X, Oh Y, Gu N (2022) Effects of oral administration of polystyrene nanoplastics on plasma glucose metabolism in mice. Chemosphere 288:132607. https://doi.org/10.1016/j.chemosphere.2021.132607

Haddadi A, Kessabi K, Boughammoura S, Rhouma MB, Mlouka R, Banni M, Messaoudi I (2022) Exposure to microplastics leads to a defective ovarian function and change in cytoskeleton protein expression in rat. Environ Sci Pollut Res Int https://doi.org/10.1007/s11356-021-18218-3

Han Y, Song Y, Kim GW, Ha C, Lee J, Kim M, Son H, Lee G, Gautam R, Heo Y (2021) No prominent toxicity of polyethylene microplastics observed in neonatal mice following intratracheal instillation to dams during gestational and neonatal period. Toxicological Research 37:443-450. https://doi.org/10.1007/s43188-020-00086-7

Hou B, Wang F, Liu T, Wang Z (2021a) Reproductive toxicity of polystyrene microplastics: In vivo experimental study on testicular toxicity in mice. J Hazard Mater 405:124028. https://doi.org/10.1016/j.jhazmat.2020.124028

Hou J, Lei Z, Cui L, Hou Y, Yang L, An R, Wang Q, Li S, Zhang H, Zhang L (2021b) Polystyrene microplastics lead to pyroptosis and apoptosis of ovarian granulosa cells via NLRP3/Caspase-1 signaling pathway in rats. Ecotoxicol Environ Saf 212:112012. https://doi.org/10.1016/j.ecoenv.2021.112012

Hu J, Qin X, Zhang J, Zhu Y, Zeng W, Lin Y, Liu X (2021) Polystyrene microplastics disturb maternal-fetal immune balance and cause reproductive toxicity in pregnant mice. Reprod Toxicol 106:42-50. https://doi.org/10.1016/j.reprotox.2021.10.002

Huang T, Zhang W, Lin T, Liu S, Sun Z, Liu F, Yuan Y, Xiang X, Kuang H, Yang B, Zhang D (2022) Maternal exposure to polystyrene nanoplastics during gestation and lactation induces hepatic and testicular toxicity in male mouse offspring. Food Chem Toxicol 160:112803. https://doi.org/10.1016/j.fct.2021.112803

Ijaz MU, Shahzadi S, Samad A, Ehsan N, Ahmed H, Tahir A, Rehman H, Anwar H (2021) Dose-Dependent Effect of Polystyrene Microplastics on the Testicular Tissues of the Male Sprague Dawley Rats. Dose Response 19:15593258211019882. https://doi.org/10.1177/15593258211019882

Jeong B, Baek JY, Koo J, Park S, Ryu YK, Kim KS, Zhang S, Chung C, Dogan R, Choi HS, Um D, Kim TK, Lee WS, Jeong J, Shin WH, Lee JR, Kim NS, Lee DY (2022) Maternal exposure to polystyrene nanoplastics causes brain abnormalities in progeny. J Hazard Mater 426:127815. https://doi.org/10.1016/j.jhazmat.2021.127815

Jiang P, Yuan GH, Jiang BR, Zhang JY, Wang YQ, Lv HJ, Zhang Z, Wu JL, Wu Q, Li L (2021) Effects of microplastics (MPs) and tributyltin (TBT) alone and in combination on bile acids and gut microbiota crosstalk in mice. Ecotoxicol Environ Saf 220:112345. https://doi.org/10.1016/j.ecoenv.2021.112345

Jin H, Ma T, Sha X, Liu Z, Zhou Y, Meng X, Chen Y, Han X, Ding J (2021) Polystyrene microplastics induced male reproductive toxicity in mice. J Hazard Mater 401:123430. https://doi.org/10.1016/j.jhazmat.2020.123430

Jin H, Yan M, Pan C, Liu Z, Sha X, Jiang C, Li L, Pan M, Li D, Han X, Ding J (2022) Chronic exposure to polystyrene microplastics induced male reproductive toxicity and decreased testosterone levels via the LH-mediated LHR/cAMP/PKA/StAR pathway. Part Fibre Toxicol 19:13. https://doi.org/10.1186/s12989-022-00453-2

Jin Y, Lu L, Tu W, Luo T, Fu Z (2019) Impacts of polystyrene microplastic on the gut barrier, microbiota and metabolism of mice. Sci Total Environ 649:308-317. https://doi.org/10.1016/j.scitotenv.2018.08.353

Jing J, Zhang L, Han L, Wang J, Zhang W, Liu Z, Gao A (2022) Polystyrene micro-/nanoplastics induced hematopoietic damages via the crosstalk of gut microbiota, metabolites, and cytokines. Environ Int 161:107131. https://doi.org/10.1016/j.envint.2022.107131

Kang K, Yang D, Huang Z, Zhang S, Fang T, Jiang Y, Wu J (2019) Effects of microplastics on the growth and structure of the mouse small intestine. J Agro-Environ Science 39(2):256-262. https://doi.org/10.11654/jaes.2019-0851

Kim J, Maruthupandy M, An KS, Lee KH, Jeon S, Kim JS, Cho WS (2021) Acute and subacute repeated oral toxicity study of fragmented microplastics in Sprague-Dawley rats. Ecotoxicol Environ Saf 228:112964. https://doi.org/10.1016/j.ecoenv.2021.112964

Kwon W, Kim D, Kim HY, Jeong SW, Lee SG, Kim HC, Lee YJ, Kwon MK, Hwang JS, Han JE, Park JK, Lee SJ, Choi SK (2022) Microglial phagocytosis of polystyrene microplastics results in immune alteration and apoptosis in vitro and in vivo. Sci Total Environ 807:150817. https://doi.org/10.1016/j.scitotenv.2021.150817

Lee CW, Hsu LF, Wu IL, Wang YL, Chen WC, Liu YJ, Yang LT, Tan CL, Luo YH, Wang CC, Chiu HW, Yang TC, Lin YY, Chang HA, Chiang YC, Chen CH, Lee MH, Peng KT, Huang CC (2022a) Exposure to polystyrene microplastics impairs hippocampus-dependent learning and memory in mice. J Hazard Mater 430:128431. https://doi.org/10.1016/j.jhazmat.2022.128431

Lee S, Kang KK, Sung SE, Choi JH, Sung M, Seong KY, Lee S, Yang SY, Seo MS, Kim K (2022b) Toxicity Study and Quantitative Evaluation of Polyethylene Microplastics in ICR Mice. Polymers-Basel 14:402. https://doi.org/10.3390/polym14030402

Li B, Ding Y, Cheng X, Sheng D, Xu Z, Rong Q, Wu Y, Zhao H, Ji X, Zhang Y (2020a) Polyethylene microplastics affect the distribution of gut microbiota and inflammation development in mice. Chemosphere 244:125492. https://doi.org/10.1016/j.chemosphere.2019.125492

Li H, Liu S, Zhang J, Chen J (2021a) Effects of polystyrene microplastics on kidney of diabetic mice. China Environ Science 42(3):1369-1378. https://doi.org/10.19674/j.cnki.issn1000-6923.20210923.003

Li S, Ma Y, Ye S, Tang S, Liang N, Liang Y, Xiao F (2021b) Polystyrene microplastics trigger hepatocyte apoptosis and abnormal glycolytic flux via ROS-driven calcium overload. J Hazard Mater 417:126025. https://doi.org/10.1016/j.jhazmat.2021.126025

Li S, Wang Q, Yu H, Yang L, Sun Y, Xu N, Wang N, Lei Z, Hou J, Jin Y, Zhang H, Li L, Xu F, Zhang L (2021c) Polystyrene microplastics induce blood-testis barrier disruption regulated by the MAPK-Nrf2 signaling pathway in rats. Environ Sci Pollut Res Int 28:47921-47931. https://doi.org/10.1007/s11356-021-13911-9

Li X, Zhang T, Lv W, Wang H, Chen H, Xu Q, Cai H, Dai J (2022) Intratracheal administration of polystyrene microplastics induces pulmonary fibrosis by activating oxidative stress and Wnt/beta-catenin signaling pathway in mice. Ecotoxicol Environ Saf 232:113238. https://doi.org/10.1016/j.ecoenv.2022.113238

Li Z, Zhu S, Liu Q, Wei J, Jin Y, Wang X, Zhang L (2020b) Polystyrene microplastics cause cardiac fibrosis by activating Wnt/beta-catenin signaling pathway and promoting cardiomyocyte apoptosis in rats. Environ Pollut 265:115025. https://doi.org/10.1016/j.envpol.2020.115025

Liang B, Huang Y, Zhong Y, Li Z, Ye R, Wang B, Zhang B, Meng H, Lin X, Du J, Hu M, Wu Q, Sui H, Yang X, Huang Z (2022) Brain single-nucleus transcriptomics highlights that polystyrene nanoplastics potentially induce Parkinson’s Disease-like neurodegeneration by causing energy metabolism disorders in mice. J Hazard Mater https://doi.org/10.1016/j.jhazmat.2022.128459

Liang B, Zhong Y, Huang Y, Lin X, Liu J, Lin L, Hu M, Jiang J, Dai M, Wang B, Zhang B, Meng H, Lelaka JJJ, Sui H, Yang X, Huang Z (2021) Underestimated health risks: polystyrene micro- and nanoplastics jointly induce intestinal barrier dysfunction by ROS-mediated epithelial cell apoptosis. Part Fibre Toxicol 18:20. https://doi.org/10.1186/s12989-021-00414-1

Liu Z, Zhuan Q, Zhang L, Meng L, Fu X, Hou Y (2022) Polystyrene microplastics induced female reproductive toxicity in mice. J Hazard Mater 424:127629. https://doi.org/10.1016/j.jhazmat.2021.127629

Lu K, Lai KP, Stoeger T, Ji S, Lin Z, Lin X, Chan TF, Fang JK, Lo M, Gao L, Qiu C, Chen S, Chen G, Li L, Wang L (2021) Detrimental effects of microplastic exposure on normal and asthmatic pulmonary physiology. J Hazard Mater 416:126069. https://doi.org/10.1016/j.jhazmat.2021.126069

Lu L, Wan Z, Luo T, Fu Z, Jin Y (2018) Polystyrene microplastics induce gut microbiota dysbiosis and hepatic lipid metabolism disorder in mice. Sci Total Environ 631-632:449-458. https://doi.org/10.1016/j.scitotenv.2018.03.051

Luo T, Wang C, Pan Z, Jin C, Fu Z, Jin Y (2019a) Maternal Polystyrene Microplastic Exposure during Gestation and Lactation Altered Metabolic Homeostasis in the Dams and Their F1 and F2 Offspring. Environ Sci Technol 53:10978-10992. https://doi.org/10.1021/acs.est.9b03191

Luo T, Zhang Y, Wang C, Wang X, Zhou J, Shen M, Zhao Y, Fu Z, Jin Y (2019b) Maternal exposure to different sizes of polystyrene microplastics during gestation causes metabolic disorders in their offspring. Environ Pollut 255:113122. https://doi.org/10.1016/j.envpol.2019.113122

Marlene S, Niechcial A, Lee SS, Sinnet B, Wawrzyniak M, Laimbacher A, Atrott K, Manzini R, Morsy Y, Häfliger J, Lang S, Rogler G, Kaegi R, Scharl M, Spalinger MR (2022) Ingested nano- and microsized polystyrene particles surpass the intestinal barrier and accumulate in the body. NanoImpact 25 https://doi.org/10.1016/j.impact.2021.100374

Meng X, Zhang J, Wang W, Gonzalez-Gil G, Vrouwenvelder JS, Li Z (2022) Effects of nano- and microplastics on kidney: Physicochemical properties, bioaccumulation, oxidative stress and immunoreaction. Chemosphere 288:132631. https://doi.org/10.1016/j.chemosphere.2021.132631

Mu Y, Sun J, Li Z, Zhang W, Liu Z, Li C, Peng C, Cui G, Shao H, Du Z (2022) Activation of pyroptosis and ferroptosis is involved in the hepatotoxicity induced by polystyrene microplastics in mice. Chemosphere 291:132944. https://doi.org/10.1016/j.chemosphere.2021.132944

Park EJ, Han JS, Park EJ, Seong E, Lee GH, Kim DW, Son HY, Han HY, Lee BS (2020) Repeated-oral dose toxicity of polyethylene microplastics and the possible implications on reproduction and development of the next generation. Toxicol Lett 324:75-85. https://doi.org/10.1016/j.toxlet.2020.01.008

Qiao J, Chen R, Wang M, Bai R, Cui X, Liu Y, Wu C, Chen C (2021) Perturbation of gut microbiota plays an important role in micro/nanoplastics-induced gut barrier dysfunction. Nanoscale 13:8806-8816. https://doi.org/10.1039/d1nr00038a

Rafiee M, Dargahi L, Eslami A, Beirami E, Jahangiri-Rad M, Sabour S, Amereh F (2018) Neurobehavioral assessment of rats exposed to pristine polystyrene nanoplastics upon oral exposure. Chemosphere 193:745-753. https://doi.org/10.1016/j.chemosphere.2017.11.076

Rawle DJ, Dumenil T, Tang B, Bishop CR, Yan K, Le TT, Suhrbier A (2022) Microplastic consumption induces inflammatory signatures in the colon and prolongs a viral arthritis. Sci Total Environ 809:152212. https://doi.org/10.1016/j.scitotenv.2021.152212

Shengchen W, Jing L, Yujie Y, Yue W, Shiwen X (2021) Polystyrene microplastics-induced ROS overproduction disrupts the skeletal muscle regeneration by converting myoblasts into adipocytes. J Hazard Mater 417:125962. https://doi.org/10.1016/j.jhazmat.2021.125962

Stock V, Bohmert L, Lisicki E, Block R, Cara-Carmona J, Pack LK, Selb R, Lichtenstein D, Voss L, Henderson CJ, Zabinsky E, Sieg H, Braeuning A, Lampen A (2019) Uptake and effects of orally ingested polystyrene microplastic particles in vitro and in vivo. Arch Toxicol 93:1817-1833. https://doi.org/10.1007/s00204-019-02478-7

Sun H, Chen N, Yang X, Xia Y, Wu D (2021a) Effects induced by polyethylene microplastics oral exposure on colon mucin release, inflammation, gut microflora composition and metabolism in mice. Ecotoxicol Environ Saf 220:112340. https://doi.org/10.1016/j.ecoenv.2021.112340

Sun R, Xu K, Yu L, Pu Y, Xiong F, He Y, Huang Q, Tang M, Chen M, Yin L, Zhang J, Pu Y (2021b) Preliminary study on impacts of polystyrene microplastics on the hematological system and gene expression in bone marrow cells of mice. Ecotoxicol Environ Saf 218:112296. https://doi.org/10.1016/j.ecoenv.2021.112296

Tian K, Chen Q (2020) The effect of sub-acute exposure of microplastics on the male mice reproductive function. J Toxicol 34(6):492-496. https://doi.org/10.16421/j.cnki.1002-3127.2020.06.013

Wang Y, Wang S, Xu T, Cui W, Shi X, Xu S (2022) A new discovery of polystyrene microplastics toxicity: The injury difference on bladder epithelium of mice is correlated with the size of exposed particles. Sci Total Environ 821:153413. https://doi.org/10.1016/j.scitotenv.2022.153413

Wang YL, Lee YH, Hsu YH, Chiu IJ, Huang CC, Huang CC, Chia ZC, Lee CP, Lin YF, Chiu HW (2021) The Kidney-Related Effects of Polystyrene Microplastics on Human Kidney Proximal Tubular Epithelial Cells HK-2 and Male C57BL/6 Mice. Environ Health Perspect 129:57003. https://doi.org/10.1289/EHP7612

Wei J, Wang X, Liu Q, Zhou N, Zhu S, Li Z, Li X, Yao J, Zhang L (2021a) The impact of polystyrene microplastics on cardiomyocytes pyroptosis through NLRP3/Caspase-1 signaling pathway and oxidative stress in Wistar rats. Environ Toxicol 36:935-944. https://doi.org/10.1002/tox.23095

Wei Y, Zhou Y, Long C, Wu H, Hong Y, Fu Y, Wang J, Wu Y, Shen L, Wei G (2021b) Polystyrene microplastics disrupt the blood-testis barrier integrity through ROS-Mediated imbalance of mTORC1 and mTORC2. Environ Pollut 289:117904. https://doi.org/10.1016/j.envpol.2021.117904

Wei Z, Wang Y, Wang S, Xie J, Han Q, Chen M (2022) Comparing the effects of polystyrene microplastics exposure on reproduction and fertility in male and female mice. Toxicology 465:153059. https://doi.org/10.1016/j.tox.2021.153059

Xiao J, Jiang X, Zhou Y, Sumayyah G, Zhou L, Tu B, Qin Q, Qiu J, Qin X, Zou Z, Chen C (2022) Results of a 30-day safety assessment in young mice orally exposed to polystyrene nanoparticles. Environ Pollut 292:118184. https://doi.org/10.1016/j.envpol.2021.118184

Xie X, Deng T, Duan J, Xie J, Yuan J, Chen M (2020) Exposure to polystyrene microplastics causes reproductive toxicity through oxidative stress and activation of the p38 MAPK signaling pathway. Ecotoxicol Environ Saf 190:110133. https://doi.org/10.1016/j.ecoenv.2019.110133

Xu D, Ma Y, Han X, Chen Y (2021) Systematic toxicity evaluation of polystyrene nanoplastics on mice and molecular mechanism investigation about their internalization into Caco-2 cells. J Hazard Mater 417:126092. https://doi.org/10.1016/j.jhazmat.2021.126092

Zaheer J, Kim H, Ko IO, Jo EK, Choi EJ, Lee HJ, Shim I, Woo HJ, Choi J, Kim GH, Kim JS (2022) Pre/post-natal exposure to microplastic as a potential risk factor for autism spectrum disorder. Environ Int 161:107121. https://doi.org/10.1016/j.envint.2022.107121

Zhao J, Gomes D, Jin L, Mathis SP, Li X, Rouchka EC, Bodduluri H, Conklin DJ, O'Toole TE (2022) Polystyrene bead ingestion promotes adiposity and cardiometabolic disease in mice. Ecotoxicol Environ Saf 232:113239. https://doi.org/10.1016/j.ecoenv.2022.113239

Zhao L, Shi W, Hu F, Song X, Cheng Z, Zhou J (2021) Prolonged oral ingestion of microplastics induced inflammation in the liver tissues of C57BL/6J mice through polarization of macrophages and increased infiltration of natural killer cells. Ecotoxicol Environ Saf 227:112882. https://doi.org/10.1016/j.ecoenv.2021.112882

Zheng H, Wang J, Wei X, Chang L, Liu S (2021) Proinflammatory properties and lipid disturbance of polystyrene microplastics in the livers of mice with acute colitis. Sci Total Environ 750:143085. https://doi.org/10.1016/j.scitotenv.2020.143085
